# Impact of LiBOB additive on cycle-performance degradation of lithium mono-chelated borate electrolytes: minimize the crosstalk-derived deterioration†

**DOI:** 10.1039/d3ra02381h

**Published:** 2023-09-01

**Authors:** Mikihiro Takahashi, Hayato Hesaka, Hiromori Tsutsumi, Yu Katayama

**Affiliations:** a Central Glass Co., Ltd., Applied Chemical Research Center 5254-35 Okiube, Ube Yamaguchi 755-0001 Japan; b SANKEN, Osaka University 8-1 Mihogaoka, Ibaraki Osaka 567-0047 Japan yuktym@sanken.osaka-u.ac.jp; c Graduate School of Sciences and Technology for Innovation, Yamaguchi University 2-16-1 Tokiwadai, Ube Yamaguchi 755-8611 Japan

## Abstract

Novel electrolyte systems are required to further improve the performance and ensure the safety of lithium-ion batteries. Lithium-monochelated borates with trifluoromethylated ligands are used as electrolytes for lithium-ion batteries (LIBs) with a lithium bis(oxalato)borate (LiBOB) additive. The capacity decay and extremely high resistance after the cycle test at 60 °C are dramatically suppressed by the addition of LiBOB. Half-cell measurements, X-ray photoelectron spectroscopy (XPS), and electrochemical impedance spectroscopy (EIS) suggested that the reductive decomposition products of the electrolytes at the negative electrode significantly increased the resistance at the positive electrode, which originated from the crosstalk of the decomposition species formed at the negative electrode. Further analysis confirmed the importance of the LiBOB-derived solid electrolyte interphase (SEI) at the negative electrode, which suppressed the formation of crosstalk species at the negative electrode and effectively suppressed the increase in resistance of the positive electrode. This study provides a reliable and promising approach for designing high-performance electrolytes with lithium borate and emphasizes the importance of considering the reactions occurring at both electrodes to improve battery performance.

## Abstract 2

The capacity decay and high resistance after the cycle test are improved by the LiBOB-derived solid electrolyte interphase (SEI). The SEI suppressed the formation of crosstalk species at the negative electrode and effectively reduced the increase in resistance of the positive electrode.

## Graphical abstract

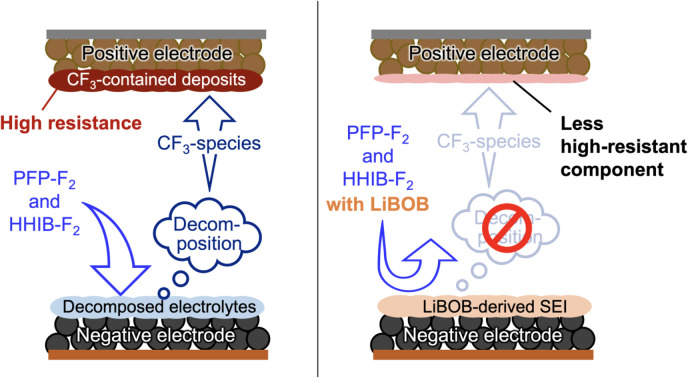

## 1. Introduction

Lithium-ion batteries (LiBs) are commonly used as power sources for mobile personal computers (PCs) and smartphones because of their light weight and high capacity.^1^ Furthermore, in recent years, automobile electrification has been aggressively pursued to reduce greenhouse gas emissions.^2,3^ In this context, the development of the large-size LiBs, having good discharge capacity, especially at low temperatures (<−10 °C),^4^ durability at high temperatures (>60 °C),^5^ and safety,^6^ is highly desired. Although lithium hexafluorophosphate (LiPF_6_) is widely used as a salt for current lithium-ion batteries,^7,8^ considering its high ionic conductivity,^9^ wide potential window,^9^ and low price,^10^ LiPF_6_ has several remaining issues, such as low thermal stability (<60 °C) as well as the formation of harmful HF *via* hydrolysis with trace water. Therefore, the development of an alternative electrolyte that can solve the abovementioned issues with LiPF_6_ remains an important and challenging task.

Lithium tetrafluoroborate (LiBF_4_) is known as a lithium salt with a better heat resistance than LiPF_6_.^11,12^ However, the ionic conductivity of LiBF_4_ is ∼5 mS cm^−1^ lower than that of LiPF_6_ (dimethylcarbonate (DMC)/ethylene carbonate (EC) = 1/1, 1 M salt at 20 °C).^9^ Recently, we successfully developed novel lithium borates, lithium difluoro(perfluoropinacolato)borate (PFP-F_2_), and lithium difluoro(2-hydroxy-3,3,3,3′,3′,3′-hexafluoroisobutylato)borate (HHIB-F_2_),^13^ which show improved ionic conductivity while maintaining thermal stability and high hydrolysis resistance. However, PFP-F_2_ and HHIB-F_2_ have been found to have a narrower potential window on the reduction side than LiBF_4_, which may decrease the battery performance by the reductive decomposition of the salt at the negative electrode. Although HHIB-F_2_ has already shown a relatively high cycle performance, further improvement in the cycle performance can be expected by suppressing reductive decomposition. Therefore, the suppression of salt decomposition is the key to achieving both high hydrolysis resistance and cycle performance.

An effective strategy for suppressing undesired reductive decomposition is to form a protective layer on the graphite surface. To form a protective layer, both *ex situ* and *in situ* methods that use graphite with a modified surface before cell assembly has been proposed.^14^ For example, oxidation treatment,^15^ coating with sodium maleate solution,^16^ and the polymerization of acrylate have been applied to the graphite surface to form a film *ex situ*,^17^ successfully suppressing electrolyte decomposition during charging and reducing the irreversible capacity. However, there are issues such as an increase in the number of processes required to assemble the cell compared to the case where an untreated active material is used. The *in situ* method forms a protective layer called a solid electrolyte interphase (SEI) on the graphite surface during the initial cycle, utilizing the reductive decomposition reaction of additives in the electrolyte.^8,18^ When using additives, it is important to select those that decompose at a higher potential than the solvents and electrolyte salts, whose decomposition must be suppressed. Common additives are vinylene carbonate (VC, decomposition potential of 0.9 V_Li_ (ref. 19)),^20,21^ 1,3,2-dioxathiolane 2,2-dioxide (DTD, decomposition potential of 1.3 V_Li_ (ref. 19))^19,22^ lithium difluoro(oxalate)borate (LiDFOB, decomposition potential of 1.6–1.7 V_Li_ (ref. 13, 23 and 24)),^25,26^ and lithium bisoxalatoborate (LiBOB, decomposition potential of 1.7–1.8 V_Li_ (ref. 13, 24 and 27)),^28,29^ all of which forms the SEI on the negative electrode surface and suppress further decomposition of the electrolytes.

Here, we selected a LiBOB additive to suppress the reductive decomposition of two novel lithium borates, PFP-F_2_ and HHIB-F_2_, to achieve high cycle performance while maintaining thermal stability and high hydrolysis resistance. The LiBOB additive was selected owing to the high decomposition potential of 1.7–1.8 V_Li_, which is suitable for suppressing PFP-F_2_ and HHIB-F_2_ with relatively high decomposition potential (narrow potential window). The capacity at the 100^th^ cycle was improved by 66.7 and 13.9%, respectively, and the resistance after the cycle test was reduced to 3.9 and 27.5%, respectively, compared to those without LiBOB additives. The capacity evaluation of the reconstructed cells with recovered negative or positive electrodes after the cycle test revealed that the positive electrode was the major cause of discharge capacity decay during the cycle test. Furthermore, electrochemical impedance spectroscopy (EIS) measurements revealed that the addition of LiBOB significantly suppressed the increase in the resistance of the positive electrode after cycling. The surface deposits on the electrode surface were probed by X-ray photoelectron spectroscopy (XPS), confirming a decrease in the CF_3_ moiety within the surface deposit, which is the decomposition product of PFP-F_2_ and HHIB-F_2_, on both the positive and negative electrodes with LiBOB addition. This result implies the existence of crosstalk reactions originating from the negative electrode, and we propose that the suppression of electrolyte reductive decomposition by the LiBOB-derived SEI at the negative electrode mitigates the electrolyte decomposition products deposited on the positive electrode side. The cycle tests using a negative electrode with a pre-formed LIBOB-derived SEI and an electrolyte without LiBOB validated our hypothesis, where we observed a significant decrease in positive electrode resistance and improved cycle capacity, which was observed with the LiBOB additive in the electrolyte. The results emphasize the significant impact of the crosstalk of the decomposition product on the cycling performance, highlighting the importance of protecting not only the electrode that shows a significant increase in resistance but also the opposite electrode from electrolyte decomposition.

## 2. Experimental

### 2.1. Electrolyte synthesis

**Fig. 1:**
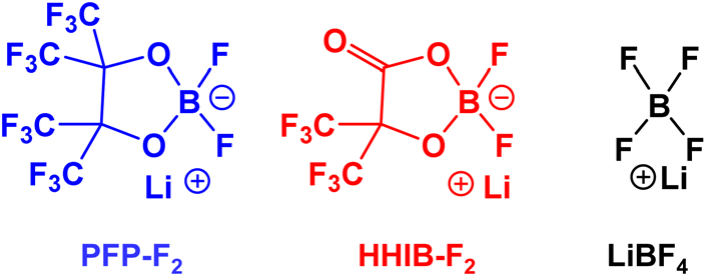
Lithium borates used in this study.

Fig. 1 shows the structures of the Li borates studied herein. Lithium difluoro(perfluoropinacolato)borate (PFP-F_2_) was synthesized using the following procedure: perfluoropinacol (47.5 g, 142.3 mmol) (Tokyo Chemical Industry, Japan) and LiBF_4_ (Battery grade, Kishida Chemical, 13.1 g, 139.5 mmol) were dissolved in 140.0 g of dehydrated ethyl methyl carbonate (EMC, Battery grade, Kishida Chemical). Then, 53 g of 60 wt% chlorotrimethylsilane (Tokyo Chemical Industry) EMC solution was added to the solution drop by drop at 5 °C. The mixture was stirred for 5 h at 40 °C under a nitrogen atmosphere. EMC and unreacted chlorotrimethylsilane were removed at 40 °C under vacuum conditions. The residual oil (containing 35 wt% of EMC) was dissolved in 20 g of dehydrated chloroform (FUJIFILM Wako Chemicals, Japan) and 10 g of dehydrated hexane (FUJIFILM Wako Chemicals). The precipitated LiBF_4_ was then removed from the solution by filtration. The remaining solution was vacuumed to remove the chloroform and hexane. Residual oil (containing 33 wt% of EMC) was dissolved in a minimum amount of chloroform (approximately 8 g), and 136 g of hexane was added to the solution. After storing for two days at −10 °C, the precipitated crystalline product was filtered at the same temperature. Subsequently, the solvent was removed under a vacuum at 40 °C. The final product was obtained as EMC adduct (74.3 g, 34 wt% of EMC). The purity was >99%, except for EMC (calculated from the peak areas of ^11^B and ^19^F NMR; Fig. S1 and S2†), and with a yield of 91%. All procedures were performed under nitrogen atmosphere. ^13^C NMR (101 MHz, CD_3_CN) *δ* 112.73 (q, *J* = 238.1 Hz, CF_3_), 83.9 (br, C̲(CF_3_)_2_); ^11^B NMR (129 MHz, CD_3_CN, LiBF_4_ = 0 [ppm]) *δ* 6.45 (s); ^19^F NMR (377 MHz, CD_3_CN, C_6_F_6_ = 0 [ppm]) *δ* 94.06 (t, *J* = 2.26 Hz, CF_3_), 16.52 (s, BF).

Lithium difluoro(2-hydroxy-3,3,3,3′,3′,3′-hexafluoroisobutylato)borate (HHIB-F_2_) was synthesized according to the following procedure: 2-hydroxy-3,3,3,3′,3′,3′-hexafluoroisobutiric acid (30.2 g, 142.3 mmol) (synthesized by the method described in the literature^30^) and LiBF_4_ (13.1 g, 139.5 mmol) was dissolved in 140 g of dehydrated EMC. Then, 53 g of 60 wt% chlorotrimethylsilane–EMC solution was added to the solution drop by drop at 5 °C. The mixture was stirred for 10 h at room temperature under a nitrogen atmosphere. EMC and unreacted chlorotrimethylsilane were removed at 25 °C under vacuum conditions. Triethylamine (Tokyo Chemical Industry) (0.23 g, 2.1 mmol) was added to neutralize the residues and hydroxyl carboxylic acid, and the mixture was stirred for 10 min at room temperature. EMC was removed at 40 °C under vacuum conditions. Dehydrated chloroform (1400 g) was added to the residual oil (containing 34 wt% of EMC). After 12 h of storage at room temperature, the precipitated product was filtered off, and then the solvent was removed under vacuum at 40 °C. The final product (24.5 g) was obtained with >99% purity (calculated from the peak area of ^11^B and ^19^F NMR; Fig. S1 and S2†) with a yield of 66%. All procedures were performed under a nitrogen atmosphere. ^13^C NMR (101 MHz, CD_3_CN) *δ* 112.09 (q, *J* = 232.2 Hz, CF_3_), 79.2 (m, *J* = 24.9 Hz, C̲(CF_3_)_2_); ^11^B NMR (129 MHz, CD_3_CN) *δ* 5.76 (s); ^19^F NMR (377 MHz, CD_3_CN) *δ* 88.11 (s, CF_3_), 15.38 (s, BF).

### 2.2. Coin cell assembly and electrochemical test

Battery-grade materials ethylene carbonate (EC), ethyl methyl carbonate (EMC), LiBF_4_, and vinylene carbonate (VC) were purchased from Kishida Chemical (Japan). The electrolyte was prepared by mixing lithium salts (PFP-F_2_, HHIB-F_2_, and LiBF_4_) with EC/EMC (1/2 by volume) solvent. Subsequently, 1 wt% LiBOB additive was introduced to the electrolyte to clarify the effect of additives. The salt concentration was set to 1.0 M for PFP-F_2_, HHIB-F_2_, and LiBF_4_. The composite NMC111 positive electrode was prepared by mixing 90.2 wt% LiNi_0.33_Mn_0.33_Co_0.33_O_2_ active material (MX-6; Umicore, Belgium), 3.8 wt% conductive carbon (HS-100; Denka, Japan), and 6.0 wt% polyvinylidene fluoride (PVDF) binder (L#7208; Kureha, Japan). The composite graphite negative electrode contained 90.0 wt% graphite (MAG-D; Hitachi Chemical, Japan) along with 10.0 wt% PVDF binder (L#9130; Kureha, Japan). For the lithium metal negative electrode, a 0.1 mm thick rolled lithium foil purchased from Honjo Metal (Japan) was used. The current collectors were Cu foil (10 μm thick, A1085) and Al foil (20 μm thick) manufactured by UACJ Foil Corporation (Japan). The cellulose separator (TF40-30) was purchased from Nippon Kodoshi (Tokyo, Japan). The active mass loading of the positive and negative electrodes was 12.2 and 6.7 mg cm^−2^, respectively. 2032-type coin cells were built with NMC111 positive electrode (*d* = 10.0 mm), graphite negative electrode (*d* = 12.0 mm), separator (*d* = 16.0 mm), and 40 μL of electrolytes with and without additives. Coin cells were assembled in an argon-filled glove box (the content of H_2_O below 1 ppm). The theoretical capacities of the positive and negative electrodes are 1.6 and 2.7 mA h, respectively (ratio of N/P is 1.7), and the current values were set based on the capacity of the positive electrode. The charge and discharge capacities were calculated based on the weight of the LiNi_0.33_Mn_0.33_Co_0.33_O_2_ active material. During the pre-charge/discharge, the cells were charged at a constant current rate of 0.2C (0.32 mA) to 4.3 V (constant current/constant voltage mode) at 25 °C, left to rest for 1 h, and discharged in constant current mode (0.2C) to 3.0 V at 25 °C. During the subsequent cycle (100 cycles), the cells were charged to 4.3 V in constant current/constant voltage mode at 3C (4.86 mA) and 60 °C, left to rest for 1 min at 60 °C, and discharged to 3.0 V in constant current mode at 3C and 60 °C. After the pre-charge/discharge (*i.e.*, before the cycle) and after the cycle, the cells were probed by electrochemical impedance spectroscopy (EIS; ALS-660C electrochemical analyzer, BAS, Japan) at 25 °C and a state of charge (SOC) of 100% (25 °C, 0.2C charging) using an amplitude of 10 mV and a frequency range of 100 kHz to 10 mHz (Schemes S1, S3, and S4†). Cyclic voltammetry (CV) was performed at 25 °C on an electrochemical analyzer (ALS-604E, BAS, Japan) at a scanning rate of 1 mV s^−1^. Lithium half-cells with graphite or NMC111 as the working electrode were used for the measurements.

### 2.3. Post-cycle test analysis

After EIS measurements, the cells were discharged at 3.0 V and disassembled in an argon-filled glove box (the content of H_2_O below 1 ppm). The recovered electrodes were immersed in 1 mL anhydrous EMC for 30 min at ambient temperature twice to remove adhered residual electrolyte and their decomposition products, subsequently vacuum-dried for 2 h at 25 °C. The dried electrodes were used for XPS and SEM measurements or electrodes for the reconstruction cell.

### 2.4. Analytical method and surface analysis of the electrodes

^1^H, ^11^B, ^13^C, and ^19^F NMR spectra were used to check the purity, and the decomposition products were recorded using a JEOL JNM-ECZ400S spectrometer in deuterated acetonitrile (CD_3_CN, Tokyo Chemical Industry, Japan) at ambient temperature. After the cycle test, cells were disassembled in an argon-filled glove box (the content of H_2_O below 1 ppm). The recovered positive and negative electrodes, and separator were immersed in 0.5 mL of CD_3_CN for 1 hour at ambient temperature. The CD_3_CN solution was then used to measure ^11^B NMR spectra. XPS analysis was performed with a PHI 5000 VersaProbe II (ULVAC-PHI, Japan) system using Al Kα radiation (*hv* = 1486.6 eV) and a charge neutralizer under ultrahigh vacuum conditions. All the electrodes were transferred from the glovebox to the XPS chamber using a transfer vessel to avoid contact with air. The adventitious hydrocarbon peak (284.3 eV) was used to calibrate all the XPS spectra. The obtained spectra were analyzed using Multipack software (ver. 9.6.0.15). After subtracting the Shirley-type background, the XPS spectra were fitted using Gaussian–Lorentzian (80 : 20) functions.

## 3. Results and discussion

### 3.1. Cycle performance

**Fig. 2:**
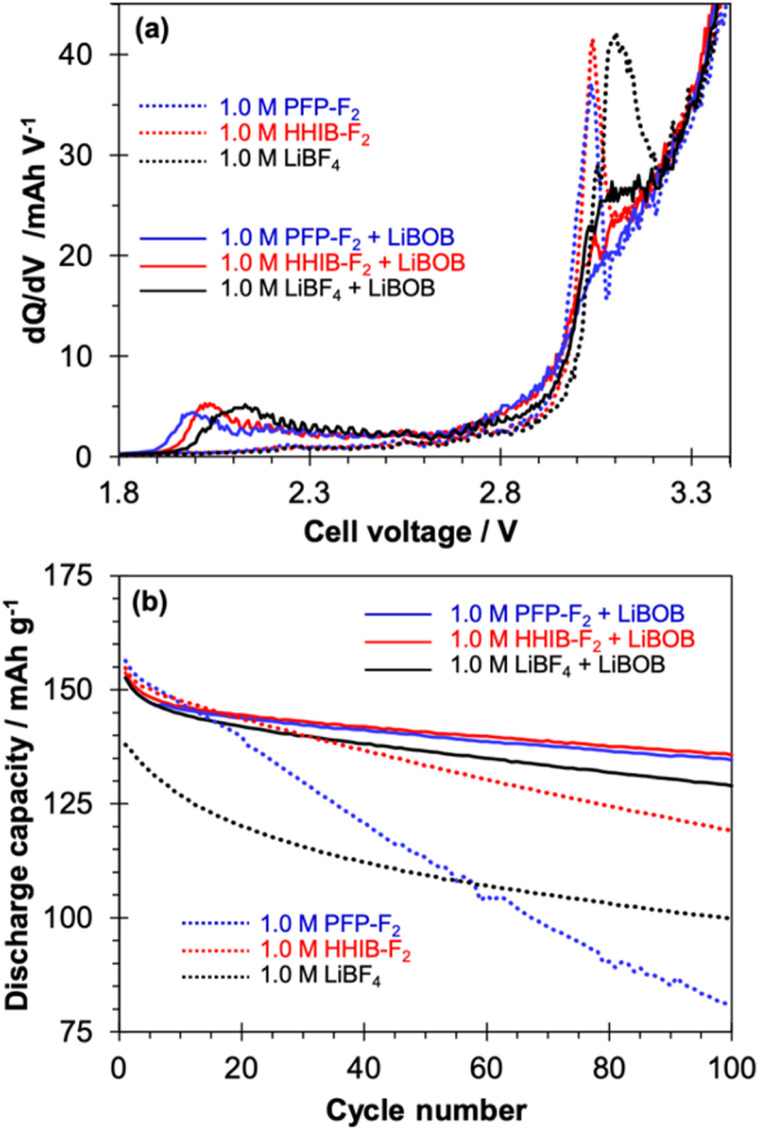
(a) Differential capacity profiles at pre-charge from LiNi_0.33_Mn_0.33_Co_0.33_O_2_/graphite cells at 0.2C rate (25 °C). The electrolytes contain 1.0 M of lithium borate, PFP-F_2_ (blue), HHIB-F_2_ (red), and LiBF_4_ (black). The dotted and solid lines show the electrolyte solution without and with 1 wt% LiBOB, respectively (solvent: EC/EMC = 1/2 v). (b) Cycle performance of full cells with corresponding electrolyte without (dotted) and with (solid) 1 wt% LiBOB (60 °C, 3C rate within a voltage range of 3.0 V to 4.3 V).

To explore the effect of the potential of LiBOB additive on PFP-F_2_, HHIB-F_2_, and LiBF_4_ electrolytes, cycle test was conducted (Fig. 2). The addition of 1 wt% LiBOB dramatically improved a discharge capacity fading observed in PFP-F_2_ and HHIB-F_2_, leading to superior cyclability than LiBF_4_.

Cycle tests were performed on graphite/NMC111 cells with PFP-F_2_, HHIB-F_2_, and LiBF_4_ electrolytes, with and without 1.0 wt% LiBOB additives at 60 °C and 3C, according to the procedure in Scheme S1.† A distinct peak was observed at *ca.* 3.1 V in the d*Q*/d*V* profiles at pre-charging (Fig. 2a), corresponding to the reductive decomposition of EC.^21,22^ Reductive decomposition of salt (LiBF_4_,^13,24^ PFP-F_2_,^13^ and HHIB-F_2_ (ref. 13)) also occurs at 0.7–1.1 V; thus the peaks around 3.0–3.1 V seen in the LiBOB-free electrolyte (dotted line) may also suggest the progress of reductive decomposition of the salts. In contrast, the d*Q*/d*V* profile of the electrolyte containing LiBOB (solid line) significantly differed from that without LiBOB in the appearance of a new peak around 2.0 V, together with a significant decrease in the reductive decomposition peaks of the electrolytes at approximately 3.0–3.1 V. The peak around 2.0 V was attributed to the reductive decomposition of LiBOB,^21,31,32^ suggesting that LiBOB was reductively decomposed in the electrolyte tested in this study. The decrease in the peak around 3.0–3.1 V indicates the suppressed decomposition of the electrolyte. The effect of the addition of LiBOB on the discharge capacity transition was also observed during the cycling test (Fig. 2b). Without LiBOB (dotted line), differences in the discharge capacity were observed in the first cycle for the PFP-F_2_ (156.4 mA h g^−1^), HHIB-F_2_ (154.8 mA h g^−1^), and LiBF_4_ (138.0 mA h g^−1^) electrolytes. In contrast, the discharge capacities at the first cycle of the electrolyte with LiBOB (solid line) were 152.6, 153.7, and 152.7 mA h g^−1^ for PFP-F_2_, HHIB-F_2_, and LiBF_4_, respectively. Although the discharge capacities of HHIB-F_2_ and PFP-F_2_ decreased slightly, a significant increase was observed for LiBF_4_ with the addition of LiBOB. The rate of discharge capacity decay in HHIB-F_2_ and LiBF_4_ without LiBOB (dotted line) slowed down at approximately 5 and 30 cycles, respectively, whereas in PFP-F_2_, the discharge capacity decreased linearly until the end of the experiment. The coulombic efficiency shown in Fig. S3† confirms that LiBF_4_ reaches a coulombic efficiency of *ca.* 99.5% after approximately 30 cycles, whereas the coulombic efficiency of PFP-F_2_ is not stable throughout the cycles, suggesting the progress of side reactions. The discharge capacities after 100 cycles with/without LiBOB were 80.7/134.6, 119.1/135.7, and 99.9/128.9 mA h g^−1^ for PFP-F_2_, HHIB-F_2_, and LiBF_4_, respectively. The addition of LiBOB clearly suppressed the discharge capacity decay for all the electrolytes tested, and the largest improvement was seen for PFP-F_2_ (53.9 mA h g^−1^ improvement). Note that the capacity improvement was the largest in the case of adding 1.0 and 1.5 wt% of LiBOB, and less significant for 0.5 wt% (Fig. S4†). Therefore, we concluded that the 1.0 wt% is the optimal amount of LiBOB.

The discharge capacity decay during the cycle test was not because of a single cause but owing to the interaction of several factors, including (1) a decrease in available Li^+^ due to Li^+^ consumption by the decomposition of the electrolytes,^33–36^ (2) a large overvoltage due to increased resistance from surface decomposition products,^33,36^ and (3) a loss of conductive pathways because of cracks caused by expansion and contraction of the electrode active materials.^34,35^ The cyclic voltammogram confirms that no significant electrolyte decomposition occurred at the potential range of 0–2.5 V_Li_ (Fig. S5†). Furthermore, a small reduction current was observed at 1.65 V_Li_ for all the electrolytes with LiBOB additive, corresponding to the LiBOB decomposition. The peak was only observed in the first cycle and disappeared after the second cycle suggesting the formation of SEI on the graphite negative electrode by LiBOB, which agrees with the d*Q*/d*V* profiles. Since the addition of LiBOB effectively suppressed the discharge capacity decay, (1) suppression of Li^+^ loss and (2) overvoltage decrease could be responsible for the observed improvement. It is to be noted that a reduction in polarization was observed in the charge/discharge curves for the PFP-F_2_ and HHIB-F_2_ electrolytes (Fig. S6(a)–(f)†) before and after the addition of LiBOB, suggesting a decrease in resistance upon the addition of LiBOB.

To confirm the effect of LiBOB on the resistance, electrochemical impedance spectroscopy (EIS) was performed before and after the cycle test (Fig. S7†). The resistance calculated from the size of the semicircles (after the cycle test without LiBOB (Fig. S7a and b†)) were 25/1800, 20/200, and 25/40 Ω for PFP-F_2_, HHIB-F_2_, and LiBF_4_, respectively. A significant increase was observed for PFP-F_2_ and HHIB-F_2_, which is consistent with the trend of the polarization increase observed in Fig. S6.† The resistance increase during the cycle test was significantly suppressed by adding LiBOB resulting in the values of 60/70, 40/55, and 30/35 Ω for PFP-F_2_, HHIB-F_2_, and LiBF_4_, respectively (Fig. S7c and d†). The observed suppression of the resistance increased during the cycle test for the HHIB-F_2_ and PFP-F_2_ electrolytes, indicating the suppressed deposition of the decomposition products on the electrode surface upon LiBOB addition.

**Fig. 3:**
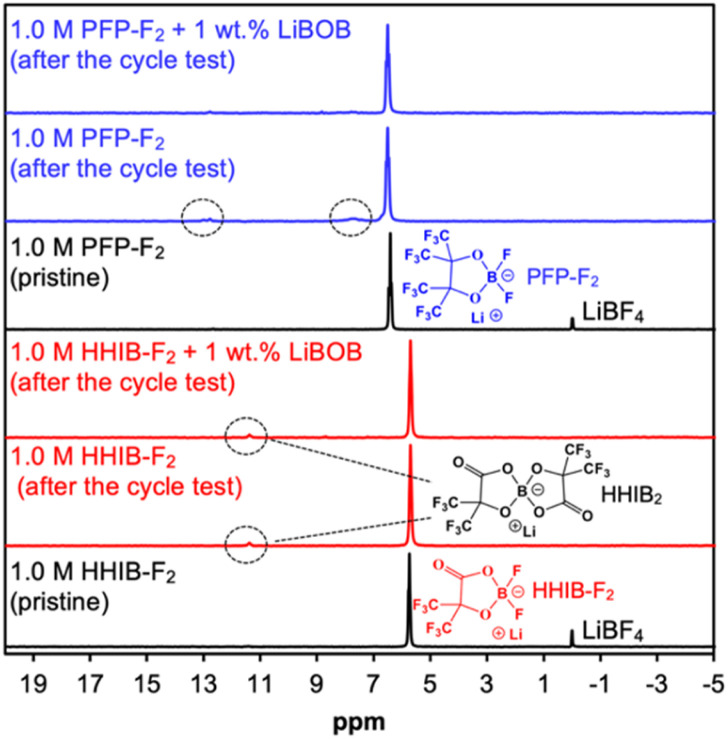
^11^B-NMR spectra of pristine electrolytes and electrolytes extracted from the cycle tested cell. LiBF_4_ at 0 ppm is an internal standard added to the pristine PFP-F_2_ and HHIB-F_2_ samples. The LiBF_4_ internal standard was not added to the electrolytes extracted from cycle tested cell to avoid the overlap with the generated LiBF_4_*via* the decomposition of PFP-F_2_ and HHIB-F_2_ during the cycle test.

The suppression of electrolyte decomposition by LiBOB addition was further confirmed by NMR analysis of the pristine electrolytes and electrolytes in the cells after the cycle test (Fig. 3).

The ^11^B-NMR spectra for the pristine PFP-F_2_ electrolyte showed only one peak at *ca.* 6.5 ppm from PFP-F_2_ (with an internal standard peak from LiBF_4_ at 0 ppm). After the cycle test without LiBOB, small additional peaks were observed at *ca.* 7.5 and 12.5 ppm, which can be attributed to the decomposition products (undetermined structure). The peaks from the decomposition products decreased in the PFP-F_2_ electrolyte after the cycle test with LiBOB, confirming the successful suppression of the decomposition reaction by the addition of LiBOB. In contrast, a different trend is observed for the HHIB-F_2_ electrolyte. Although an additional peak from the decomposition product lithium bis(2-hydroxy-3,3,3,3′,3′,3′-hexafluoroisobutylato)borate (HHIB_2_)^13^ was observed at 11.5 ppm alongside the HHIB-F_2_ peak at 5.7 ppm after the cycle test without LiBOB, the HHIB_2_ peak did not decrease in the electrolyte after the cycle test with LiBOB. These results indicated that the addition of LiBOB did not effectively suppress HHIB_2_ formation *via* HHIB-F_2_ decomposition. Considering that a clear improvement in the discharge capacity decay was observed for the HHIB-F_2_ electrolyte with LiBOB, HHIB_2_ formation had a negligible effect on cyclability.

The results thus far indicate that LiBOB effectively suppresses electrolyte decomposition, which has a negative effect on cyclability *via* (1) Li^+^ loss and (2) overvoltage increase. However, it is unclear whether the increase in resistance (owing to the deposition of decomposition products) occurs mainly on the positive or negative electrode surfaces. Therefore, we recover the electrodes after the cycle test and check the capacity and resistance of both the positive and negative electrodes, to determine the contribution of positive and negative electrodes to the significant improvement in discharge capacity and resistance after the cycle test by LiBOB addition.

### 3.2. Effect of cycling on positive and negative electrodes

**Fig. 4:**
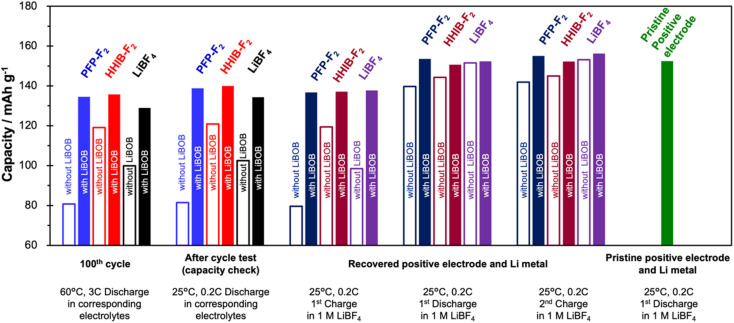
Comparison of capacities of the 100^th^ cycle, after cycle test (capacity check at 25 °C, 0.2C), and of the reconstructed half-cell (see Scheme S2† for reconstructed procedure). The capacities of left two columns (columns 1 and 2) were obtained from full cells before reconstruction, columns 3, 4, and 5 were obtained from half-cells with recovered positive electrode and Li metal, and the far right column (column 6) was obtained from a half-cell with pristine positive electrode and Li metal. Color of the bar indicates the electrolyte used for the cycling test. Filled and open bars show the cell with and without LiBOB, respectively. Charge–discharge tests for reconstructed cells were conducted in the voltage range of 3.0 V to 4.3 V at 25 °C and 0.2C, using 1.0 M LiBF_4_ electrolyte.

Charge–discharge tests with the cells reconstructed using the electrodes that were recovered from the cells after the cycle test revealed that the positive electrode was the major cause of the discharge capacity decay during the cycle test (Fig. 4).

Electrodes were recovered from the cells after the cycle test (100 cycles) in six different electrolytes (PFP-F_2_, HHIB-F_2_, LiBF_4_, PFP-F_2_ + LiBOB, HHIB-F_2_ + LiBOB, and LiBF_4_ + LiBOB), which are hereafter referred to as the recovered positive and negative electrodes. The recovered positive/negative electrode was combined with a lithium metal electrode to construct the test half-cell (see Schemes S1 and S2† for the detailed procedure).

Fig. 4 shows the charge/discharge capacities (see Fig. S8† for the corresponding charge/discharge curves) of the cell of the 100^th^ cycle in the cycle test, after the cycle test, and half-cells with recovered or pristine positive electrodes. There is no significant difference between the discharge capacity at the 100^th^ cycle in the cycle test (60 °C, 3C) and that at 25 °C, 0.2C, where charging and discharging were performed after the cycle test. The discharge capacity in a full cell after the cycle test and the 1^st^ charge capacity in a half-cell (indicating the amount of Li^+^ that can be supplied from the positive electrode) of the recovered positive electrode are also mostly identical. The similarity of these three sets of data (100^th^ cycle, after cycle test, and 1^st^ charge capacity of the recovered half-cell) suggests that the discharge capacity at the 100^th^ cycle is strongly correlated with the amount of Li^+^ that can be extracted from the recovered positive electrode. Note that the 1^st^ discharge capacity of the recovered positive electrode is notably larger than that of 1^st^ charge (with an improvement of 25–60 mA h g^−1^ for the positive electrode without LiBOB addition, and an improvement of around 15 mA h g^−1^ for the positive electrode with LiBOB addition), strongly indicates that although the amount of Li^+^ that can be extracted from the positive electrode decreases during the cycle test, the Li^+^ acceptance capacity of the positive electrode remains almost the same. In particular, the four electrolytes other than PFP-F_2_ without LiBOB and HHIB-F_2_ without LiBOB showed Li^+^ acceptance capacity (discharge capacity) equivalent to that of a pristine positive electrode (green column). The 2^nd^ charge capacity of the recovered positive electrode was clearly larger than the 1^st^ charge capacity, indicating that the amount of Li^+^ that can be extracted from the recovered positive electrode can be significantly improved by supplying a sufficient amount of Li^+^ from the lithium metal to the recovered positive electrode. Comparison of the charge–discharge capacities of the above five types of cells indicates that the main cause of the decrease in discharge capacity during cycle tests was the decrease in Li^+^ stored in the positive electrode, while the degradation of the positive electrode (decrease in the ability to insertion and deinsertion of Li^+^) was negligible. Considering that the same lithium borate salts, LiBF_4_, LiBOB, and lithium difluoro(oxalato)borate (LiDFOB), consume more than one equivalent of Li^+^ upon reductive decomposition,^24,37–39^ we believe that the decrease in Li^+^ during the cycle test is not only due to the consumption of Li^+^ during the reductive decomposition of the solvent (EC), but also can be due to the consumption of Li^+^ during the reductive decomposition of the PFP-F_2_ and HHIB-F_2_ salts. Therefore, suppressing the reductive decomposition of the electrolyte components, which is directly related to the Li^+^ consumption, by adding LiBOB effectively maintains the battery performance.

The discharge capacities of the recovered positive electrodes for PFP-F_2_ (dark blue) and HHIB-F_2_ (dark red) without LiBOB are noticeably lower than those of the other systems. Furthermore, these two systems exhibit lower discharge voltages (Fig. S8b†), indicating a larger overvoltage owing to the increased resistance. The polarization due to overvoltage is minimized, and the discharge capacity increases when the charge/discharge rate is reduced to 0.1C (Fig. S9†), further supporting our hypothesis. The same phenomenon is observed in the discharge curve of the full cell (blue and red dotted lines in Fig. S8a†), suggesting that the increase in resistance after the cycle test was strongly influenced by the positive electrode. To confirm this hypothesis, we compared the electrochemical impedance spectra of the cells with those of the recovered positive and negative electrodes after 100 cycles.

**Fig. 5:**
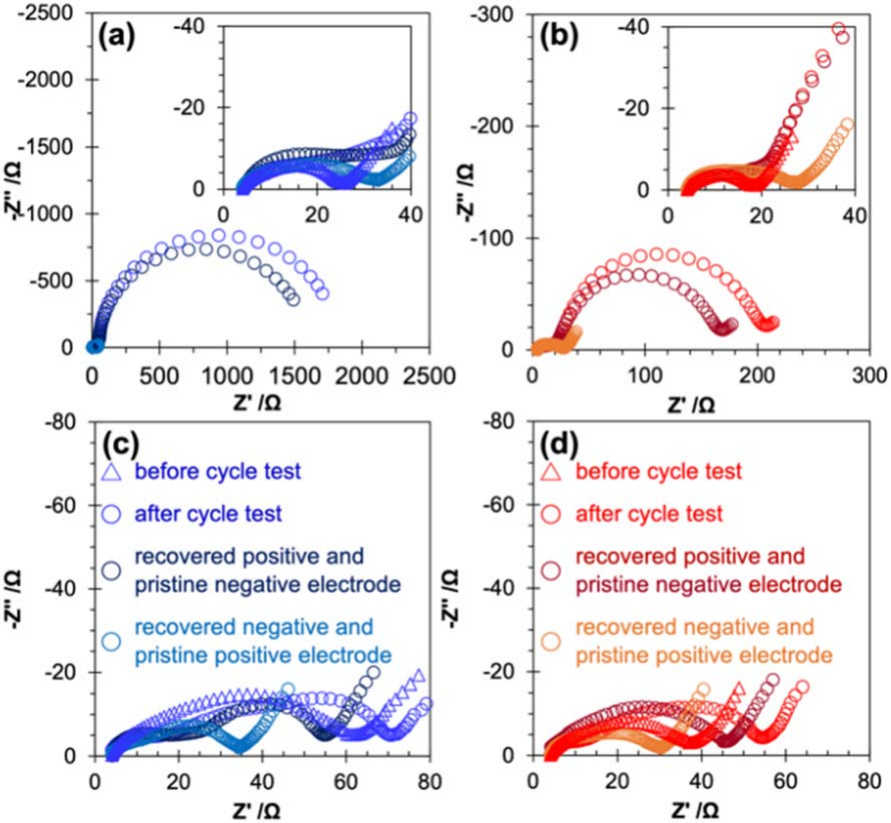
Electrochemical impedance spectra for cells with recovered and pristine electrodes in (a) PFP-F_2_, (b) HHIB-F_2_, (c) PFP-F_2_ with LiBOB, and (d) HHIB-F_2_ with LiBOB. Each legend represents the conditions of the cell for EIS measurements.

Electrochemical impedance spectroscopy confirmed a negligible change in the cell resistance of the cell with a recovered negative electrode by adding LiBOB, while a significant decrease was observed for the cell with a recovered positive electrode by adding LiBOB, suggesting that the large increase in the cell resistance after the cycle test was primarily caused by the change in the positive electrode side (Fig. 5, corresponding Bode plot in Fig. S10†).

The EIS data of the cells before and after the cycle test, and the EIS data of the reconstructed cells with recovered electrode and pristine counter electrode (recovered positive electrode and pristine negative electrode, recovered negative electrode and pristine positive electrode) are shown in Fig. 5 (see Schemes S1 and S3† for the detailed procedure). In the case of PFP-F_2_ and HHIB-F_2_ electrolytes without LiBOB (Fig. 5a and b), the cell consisting of the recovered negative and the pristine positive electrode (light blue and light red round dotes) had a semicircle diameter corresponding to 30–35 Ω, whereas the cell consisting of the recovered positive and the pristine negative electrode (dark blue and dark red round dotes) had significantly larger semicircles of about 1600 (PFP-F_2_) and 170 Ω (HHIB-F_2_). These results confirmed our hypothesis that the significant increase in cell resistance during the cycle test was mainly due to the positive electrode side.

Although there was no noticeable difference in the size of the semicircle for the cells with the recovered negative electrode with the addition of LiBOB (compare light blue in Fig. 5a and c and light red in Fig. 5b and d), the addition of LiBOB significantly reduced the size of the semicircle for the cell with the recovered positive electrode (compare dark blue in Fig. 5a and c and dark red in Fig. 5b and d). Therefore, the suppression of resistance increased after the cycle test by adding LiBOB, mainly because of the improvement on the positive electrode side. The large increase in the resistance without LiBOB and the drastic mitigation of the increase in the resistance with the addition of LiBOB were largely influenced by the deposited species on the positive electrode surface. Therefore, we performed an XPS analysis of the electrode surface to gain further insight.

### 3.3. Surface analysis of negative and positive electrodes

**Fig. 6:**
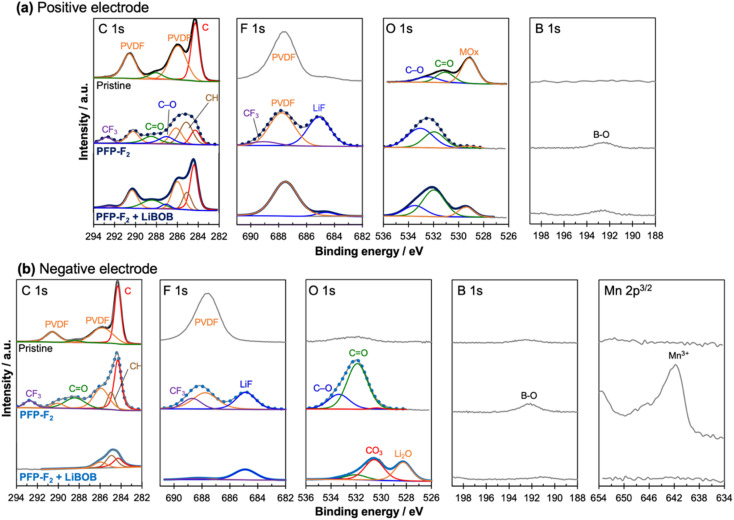
XPS spectra of a pristine and recovered (a) positive and (b) negative electrode surface after the cycle test in PFP-F_2_ electrolyte with and without LiBOB (the recovered electrodes were recovered from the cells, washed, and dried under reduced pressure before XPS measurement). From left to right, the photoemission lines for the C 1s, F 1s, O 1s, B 1s, and Mn 2p_3/2_. All spectra were calibrated with the adventitious hydrocarbons at 284.3 eV and background corrected using a Shirley background. The cycle test for recovered electrodes were performed at 25 °C, 0.2C within a voltage range of 3.0 to 4.3 V in 1.0 M PFP-F_2_ in EC/EMC with and without 1 wt% LiBOB.

XPS analysis confirmed that the addition of LiBOB to the PFP-F_2_ (Fig. 6) and HHIB-F_2_ (Fig. S11†) electrolytes significantly reduced the amount of electrolyte decomposition products in both the positive and negative electrodes, and a LiBOB-derived surface layer was observed only on the negative electrode surface.

The peak corresponding to decomposed products of PFP-F_2_ moiety were observed in each photoemission region for the cell with the recovered positive electrode (after washing with EMC and vacuum drying) cycled in PFP-F_2_ without LiBOB (Fig. 6a); CF_3_ groups at *ca.* 292.8 and 689.0 eV in the C 1s^40^ and F 1s^40,41^ spectra, respectively, lithium fluoride (LiF) at 685.0 eV in the F 1s spectrum,^25,42,43^ and the B–O bond at 192.5 eV in the B 1s spectrum.^44^ The C–O^43,45^ and C=O^43,45^ bonds were also observed at 533.2 and 532.0 eV in O 1s spectrum, originating from decomposition products of PFP-F_2_ and/or solvent. The peak of the binder-derived PVDF in the electrode is seen at 687.8 eV (ref. 25, 42 and 43) in the F 1s spectrum (the corresponding peak is buried in the C 1s spectrum). The metal oxide peak at 529.2 eV, which was visible for the pristine positive electrode in the O 1s spectrum, almost disappeared, suggesting that PFP-F_2_ and solvent decomposition products covered the surface of the positive electrode.

The CF_3_ (292.8 eV) and LiF (685.0 eV) peaks decreased significantly with the addition of LiBOB (Fig. 6a), indicating fewer decomposition products on the positive electrode surface by adding LiBOB. The visible metal oxide peak (529.2 eV) in the O 1s spectrum further supported our hypothesis. It is to be noted that the absence of additional peaks from decomposed LiBOB suggests the negligible formation of LiBOB-derived cathode electrolyte interphase (CEI). The observed trend agrees with the decrease in the positive electrode-derived cell resistance after the cycle test with the addition of LiBOB. The same trend (formation of decomposition products (LiF and CF_3_), disappearance of metal oxide peaks without LiBOB addition, and a decrease in the decomposition product peaks and visible metal oxide peaks with LiBOB addition) was observed in the HHIB-F_2_ system (Fig. S11†).

XPS analysis of the recovered negative electrode after the cycle test confirmed the formation of a LiBOB-derived SEI layer, which may protect the electrolyte components from reductive decomposition (Fig. 6b).

Similar to the results for the recovered positive electrode, peaks of CF_3_ groups (688.7, 292.8 eV; F 1s, C 1s), LiF (685.0 eV, F 1s), and B–O bonds (192.2 eV, B 1s) were detected in PFP-F_2_ without LiBOB, strongly suggesting decomposition of the salt. However, upon the addition of LiBOB (lower column), the peak intensities of the CF_3_ groups, LiF, and B–O bonds were reduced, indicating that the decomposition of the salt was suppressed. The binder-derived PVDF peaks^25,42,43^ were observed in the C 1s and F 1s spectra at 290.5, 286.1, and 687.8 eV, respectively, in the case without LiBOB, while those peaks disappeared with LiBOB. Furthermore, new lithium carbonate^46^ and lithium oxide^46^ peaks were detected in the O 1s spectra upon the addition of LiBOB, suggesting the formation of a LiBOB-derived SEI layer covering the surface of the negative electrode. Considering the possibility that PFP-F_2_-derived decomposition products were buried under the SEI, the depth profile was obtained by etching the surface (Fig. S12†). The result confirms that no CF_3_ compounds were buried on either surface; thus we concluded that the CF_3_ peak disappeared due to the suppression of reductive decomposition of PFP-F_2_. Also worth noting is that Mn was detected on the negative electrode surface only in the case without LiBOB, suggesting dissolution of Mn on the positive electrode, which diffused to the negative electrode and redeposited. Therefore, positive electrodes degrade without LiBOB, and it is one of the reasons (together with polarization) that the capacity of the positive electrode of the cells after the cycle test with PFP-F_2_ did not fully recover even after supplying Li (open dark blue bar in the third column from the right in Fig. 4). The same behavior of PFP-F_2_ and PFP-F_2_ + LiBOB on the negative electrode was also observed for HHIB-F_2_ and HHIB-F_2_ + LiBOB (Fig. S11b†).

**Fig. 7:**
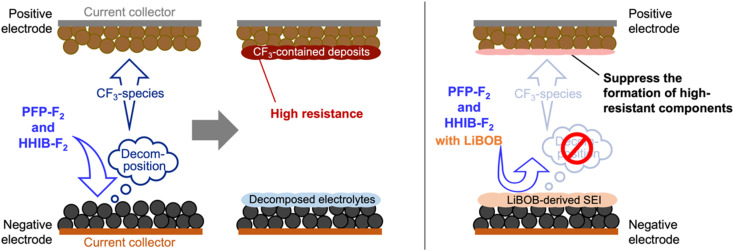
Proposed working principles for improved cycle performance observed in LiBOB-contained electrolytes. For electrolytes without LiBOB, electrolyte reductively decomposes on the negative electrode surface forming CF_3_-contained decomposition product. The CF_3_-contained decomposition product then migrate to the positive electrode side (crosstalk) and oxidatively decomposes to form highly-resistive surface layer, which has dominant role in the deterioration of the cycle performance. For LiBOB-contained system, LiBOB-derived SEI on the negative electrode surface effectively suppress the reductive decomposition of the electrolyte, leading to suppress the formation of the CF_3_-contained decomposition product. Therefore, the formation of the highly-resistive surface layer on positive electrode surface originating from the reductive decomposition product from negative electrode side is intrinsically suppressed and significantly improve the cycle performance.

Here, we summarize the results of the above analysis of the positive and negative electrodes after the cycle test and propose a working principle of LiBOB for improved cycle performance (Fig. 7).

Analysis of the discharge capacity of the various types of recovered cells using PFP-F_2_ and HHIB-F_2_ suggests that the capacity decrease observed in the cycle tests was mainly due to the loss of transferable Li^+^ and the overvoltage caused by the resistance increase(Fig. 4) and positive electrode degradation (Fig. 6b and S11b†). The loss of Li^+^ was most likely due to the consumption of Li^+^ during electrolyte decomposition (Fig. 4), and the electrolyte decomposition products caused a significant increase in the resistance on the positive electrode side (Fig. 5). The addition of LiBOB suppressed electrolyte decomposition, which moderated the loss of Li^+^ and suppressed the increase in the positive electrode resistance, resulting in an improved capacity after 100 cycles (Fig. 4). Herein, we propose that the suppression of electrolyte reductive decomposition by LiBOB-derived SEI at the negative electrode led to the mitigation of electrolyte decomposition product (CF_3_-contained deposit) on the positive electrode side (Fig. 7), considering the following: (1) PFP-F_2_ and HHIB-F_2_ are resistant to oxidation relative to reduction,^13^ (2) the electrolyte decomposition products on the positive and negative electrodes were clearly reduced by LiBOB addition (Fig. 6 and S11†), and (3) LiBOB-derived SEI of lithium carbonate and lithium oxide was formed on the negative electrode (Fig. 6b and S11b†). Our hypothesis was supported by the cycle test using a CF_3_-free binder (styrene–butadiene rubber), where we observed similar degradation without LiBOB and the improvement with LiBOB additive (Fig. S13†).

**Fig. 8:**
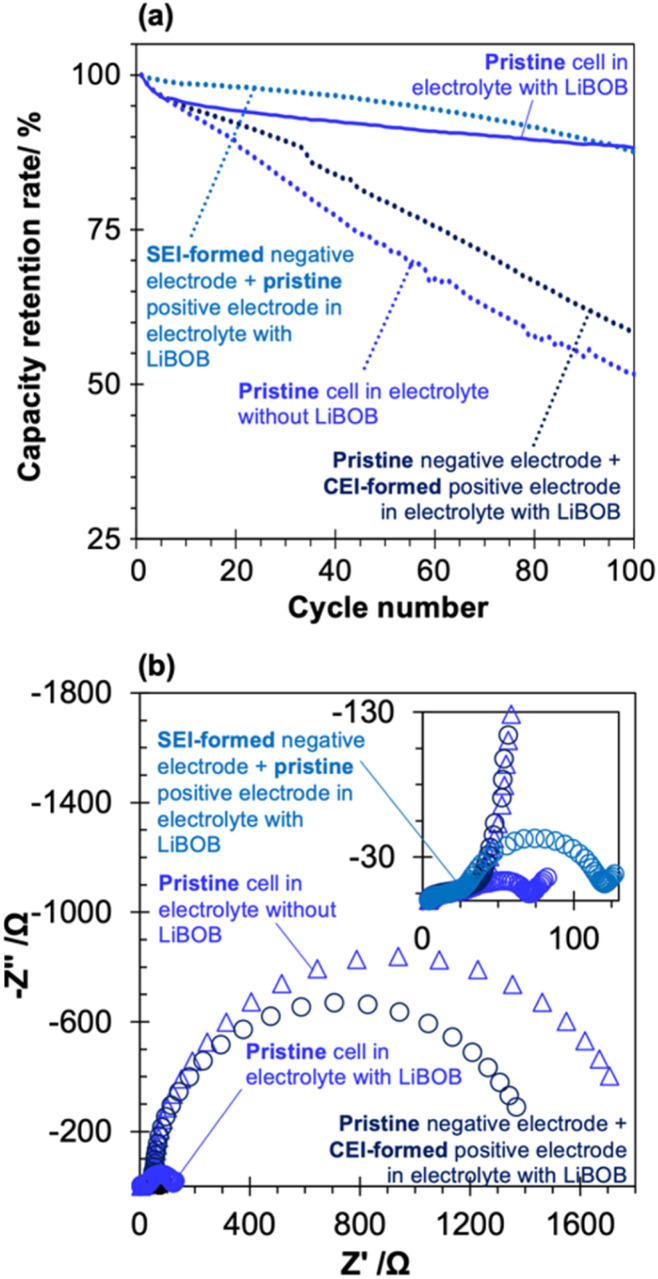
(a) Cycle retention of four different full cells. Cycle test was performed in 1.0 M PFP-F_2_ EC/EMC solution (1/2 v) with (solid line) and without LiBOB (dotted line) at 60 °C, within a voltage range of 3.0 to 4.3 V. Pre-cycling treatment to form SEI and CEI was performed in 1.0 M PFP-F_2_ EC/EMC solution (1/2 v) with LiBOB (see Scheme S4† for detailed procedure). The result from a pristine cell with (blue solid line, the same data as Fig. 2b) and without LiBOB (blue dotted line, the same data as Fig. 2b) is shown for comparison. (b) Electrochemical impedance spectra of the four types of full cells after the cycle test (corresponds to the EIS-2 in Scheme S1† and EIS-5 in Scheme S4†). Measurements were performed on cells with 100% SOC (after 0.2C charge at 25 °C) at an amplitude of 10 mV and frequency of 100 kHz to 10 mHz at 25 °C.

To further support our hypothesis, we performed a cycling test and subsequent EIS measurements in the PFP-F_2_ electrolyte without LiBOB using a cell with electrodes with a pre-SEI or pre-CEI formed in the PFP-F_2_ electrolyte with LiBOB (Fig. 8).

A comparison of the cycling results of the pristine cell (pristine negative and positive electrodes) with and without LiBOB again highlights the effect of LiBOB on cycling performance. Furthermore, cycle tests using a cell with a negative electrode with a pre-formed LiBOB-derived SEI (hereafter referred to as SEI-formed negative electrode) and a pristine positive electrode (see Scheme S4† for the detailed procedure) showed cycle performance comparable to that of the pristine cell with LiBOB, suggesting that the LiBOB-derived SEI on the negative electrode surface is the key to improved cyclability. Moreover, the cycling performance of the cell with a positive electrode with pre-formed CEI in a LiBOB-containing electrolyte (hereafter referred to as CEI-formed positive electrode; see Scheme S4† for a detailed procedure) and the pristine negative electrode showed a clear deterioration of the discharge capacity, similar to that of the pristine cell without LiBOB, further emphasizing the critical role of the LiBOB-derived SEI on the negative electrode surface (and the negligible effect of CEI on the positive electrode surface) on the cycling performance.

The EIS data obtained after the cycling test further supported this hypothesis (Fig. 8b, corresponding Bode plot in Fig. S14†). The cell with the SEI-formed negative electrode (light blue round marks) has a semi-circle size of 120 Ω, which was comparable to that of the pristine cell with LiBOB (80 Ω), while the cell with the CEI-formed positive electrode (dark blue round marks) has an extremely large semi-circle of about 1400 Ω, which was comparable to that of the pristine cell without LiBOB (∼1800 Ω). This result clearly suggests that the significant contribution to the reduction in cell resistance originates from the LiBOB-derived SEI formed on the negative electrode surface, not the CEI formed on the positive electrode. As the results in Fig. 6 indicate, the increase in cell resistance is mainly on the positive electrode side, and the results in Fig. 8b prove that the suppression of electrolyte decomposition on the negative electrode side directly affects the decrease in resistance on the positive electrode side, which supports our proposed mechanism (Fig. 8). The addition of LiBOB suppressed Mn dissolution from the positive electrode (Fig. 6b and S11b†), and a comparison of the effects of SEI and CEI derived from LiBOB indicates that SEI was more effective in suppressing Mn dissolution (Fig. S15†). The observation suggests that the decomposition products of PFP-F_2_ on the negative electrode caused the dissolution of Mn from the positive electrode. The decomposition products in the cell not only affect the electrode at which the decomposition reaction occurs but also migrate and affect the counter electrode, a phenomenon known as crosstalk.^47^ Several reports of crosstalk caused by the oxidation of the positive electrode have been published, such as metals eluted from the positive electrode deposited on the negative electrode and degrading the negative electrode,^48^ and the decomposition products of solvents on the positive electrode deposited on the negative electrode.^49^ However, there are few detailed studies on negative electrode origin cases, such as the report that there is a large correlation between deposits on the negative and positive electrode surfaces in half-cells using Li metal, but no correlation exists between deposits on the negative and positive electrode surfaces in full cells using graphite.^50^ However, our results show that in the case of PFP-F_2_ and HHIB-F_2_, a clear negative electrode-induced crosstalk occurs, significantly affecting battery performance.

## 4. Conclusions

Here, we clarify the effect of LiBOB additives on battery performance in novel electrolyte systems with the lithium borate salts PFP-F_2_ and HHIB-F_2_, on battery performances.

In cells with 1.0 M PFP-F_2_ and HHIB-F_2_ solutions (EC/EMC = 1/2), the resistance significantly increased during the cycle test at 60 °C, but the addition of LiBOB not only significantly reduced the resistance but also suppress the capacity decay. Separate analysis of the positive and negative electrodes showed that the decrease in capacity without LiBOB was mainly caused by the loss of Li^+^ owing to the decomposition of the electrolyte salts, and the increase in resistance was mainly because of the positive electrode side.

Further analysis revealed that the LiBOB additive suppressed the decomposition of electrolyte salts at the negative electrode, resulting in an increased capacity and suppression of the increase in positive electrode resistance. The cycle test without the LiBOB additive, using a negative electrode with a preformed LiBOB-derived SEI, showed a trend similar to that observed with the LiBOB additive. Therefore, the suppression of the reductive decomposition of the salt on the negative electrode side is directly related to the suppression of resistance on the positive electrode side, strongly suggesting the influence of the crosstalk of decomposed species originating at the negative electrode on the positive electrode resistance. The results highlight that not only the reaction on the positive electrode but also that on the negative electrode side must be considered to reduce the positive electrode resistance, and *vice versa*. This work clarifies the effect of electrolyte decomposition on overall cell performance, emphasizing the importance of designing a protective layer on both electrodes.

## Conflicts of interest

There are no conflicts to declare.

## Supplementary Material

RA-013-D3RA02381H-s001
