# Supplementary material for: Impact of LiBOB additive on cycle-performance degradation of lithium mono-chelated borate electrolytes: minimize the crosstalk-derived deterioration
Source: RSC Adv. 2023 Sep 1;13(37):25948–58. doi: 10.1039/d3ra02381h (PMC10472978; doi:10.1039/d3ra02381h)
Supplement: RA-013-D3RA02381H-s001 [file RA-013-D3RA02381H-s001.pdf]

Electronic Supplementary Material (ESI) for RSC Advances.
This journal is © The Royal Society of Chemistry 2023

Supporting Information

Impact of LiBOB additive on cycle-performance degradation of lithium mono-chelated borate electrolytes: minimize the crosstalk-derived deterioration

Mikihiro Takahashi [a,c], Hayato Hesaka [a], Hiromori Tsutsumi[c] , Yu Katayama [b]*

a CENTRAL GLASS CO., LTD. Applies chemical research center, 5254-35 Okiube, Ube, Yamaguchi 755-0001, Japan.

b SANKEN (The Institute of Scientific and Industrial Research), Osaka University, Mihogaoka, Ibaraki 567-0047, Osaka, Japan

c Graduate School of Sciences and Technology for Innovation, Yamaguchi University, 2-16-1 Tokiwadai, Ube, Yamaguchi 755-8611, Japan.

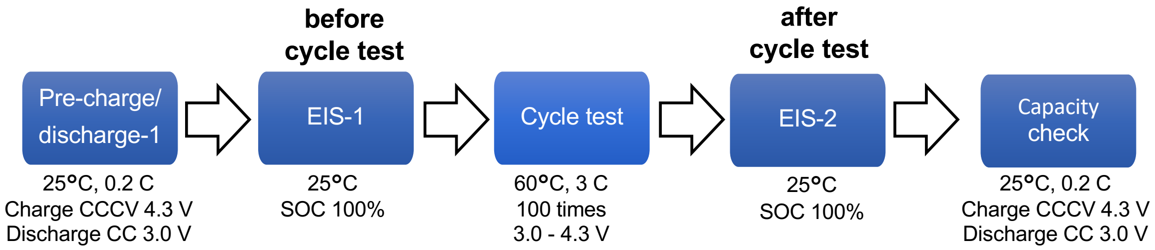

Scheme S1. Cycle test procedure

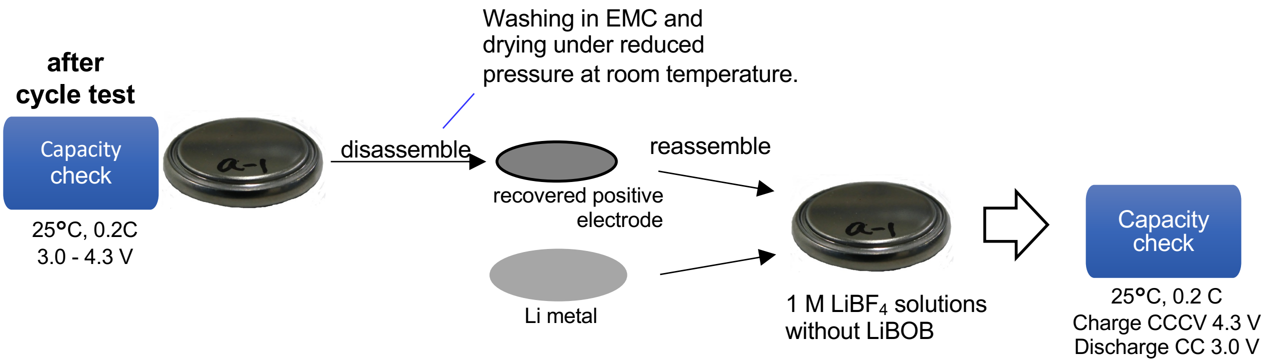

Scheme S2. Separation procedures and combination patterns of recovered electrodes to check the capacity of the positive electrode in a half-cell..

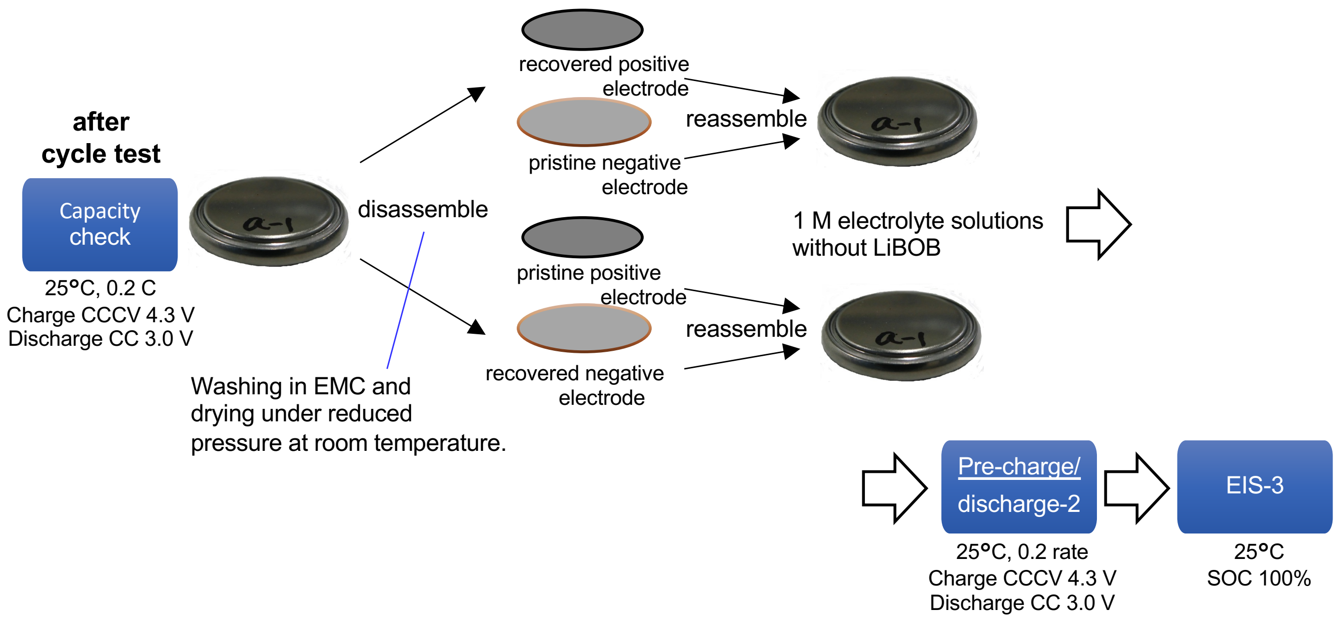

Scheme S3. Separation procedures and combination patterns of recovered electrodes to check the resistance of positive and negative electrodes.

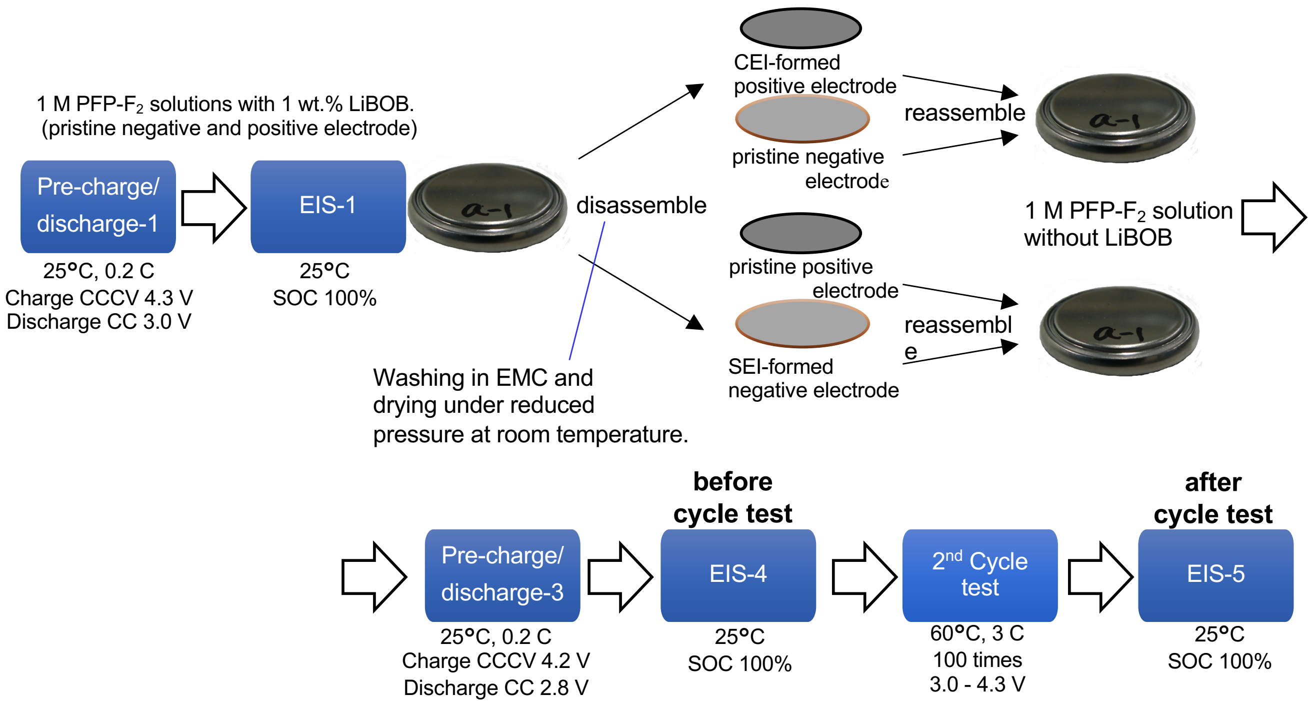

Scheme S4. Reassemble procedure of SEI/CEI-formed electrodes and the 2nd cycle test conditions.

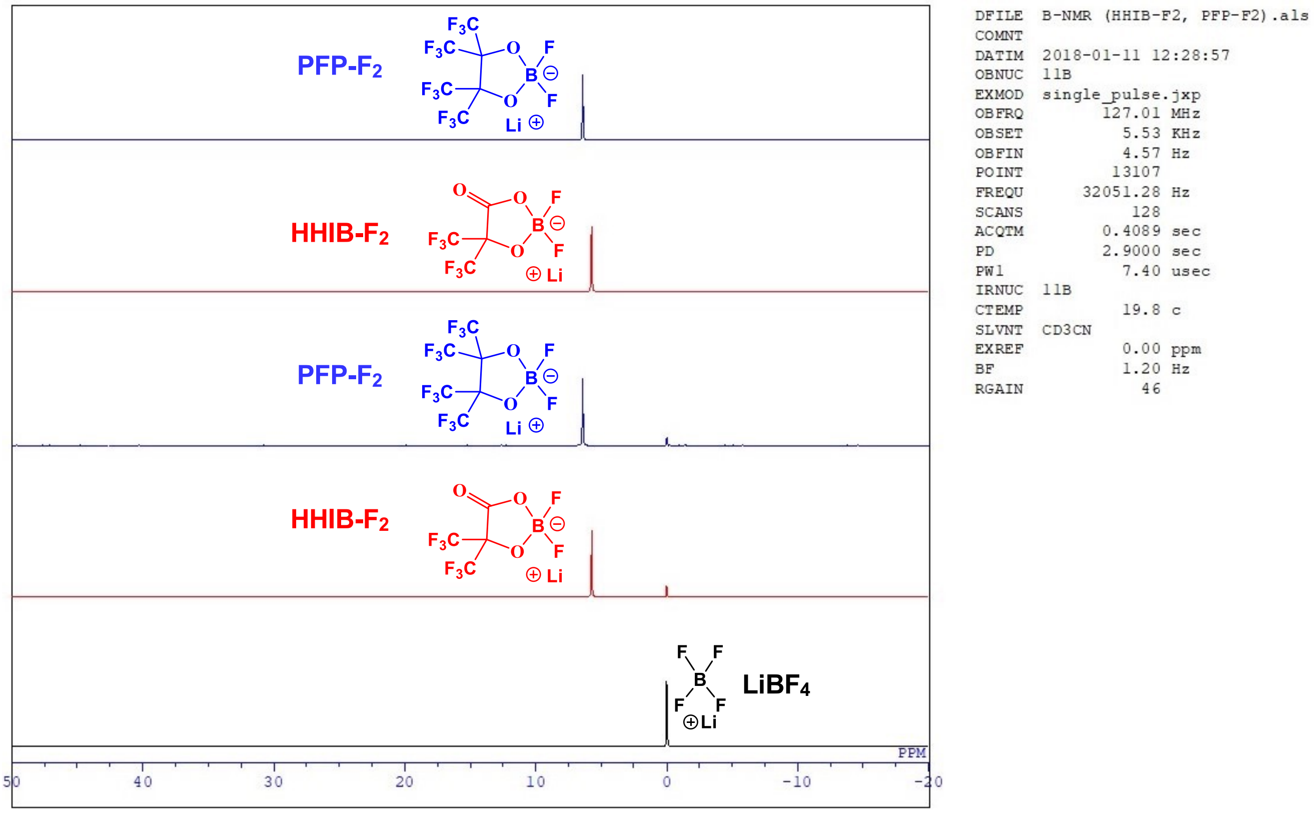

Figure S1. ^{11}B NMR spectra for each borate. To check purity, the first and second columns from the top were measured without the addition of $LiBF_4$ as a standard. (The starting material, $LiBF_4$, can be included as an impurity.) The third and fourth columns from the top are spectra obtained by adding the standard $LiBF_4$.

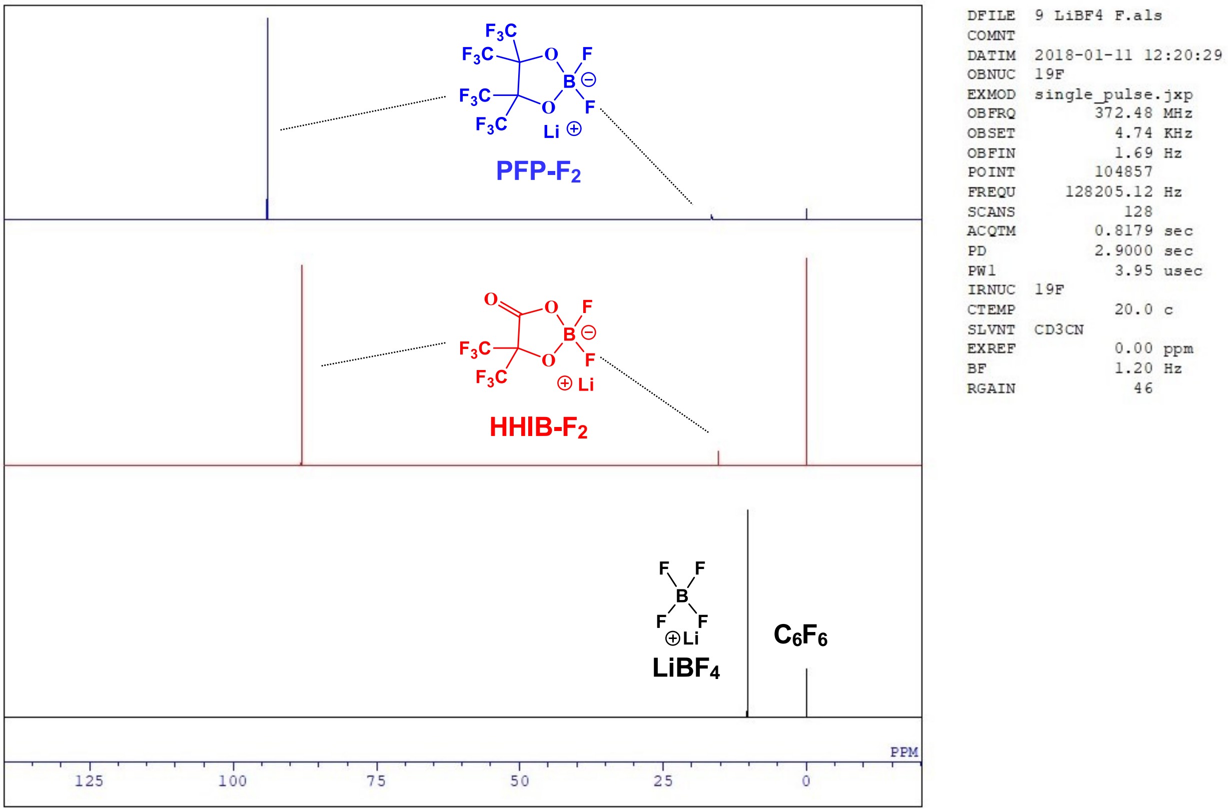

Figure S2. ^{19}F NMR chart for each borate. The C_6F_6 peak was set to 0.00 ppm.

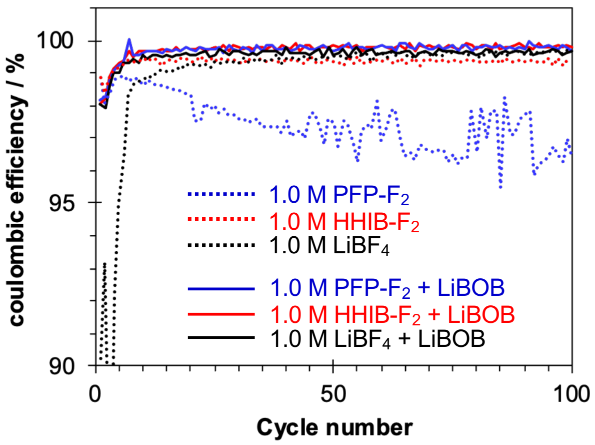

Figure S3 Coulombic efficiency in cycle test with three different electrolyte salts. The blue shows PFP-F_2 and red shows HHIB-F_2, and the black shows $LiBF_4$. The dotted lines show the electrolyte solution without LiBOB, and the solid lines show the electrolyte solution with 1 wt.% LiBOB. (60°C, 3 C rate within a voltage range of 3.0 to 4.3 V).

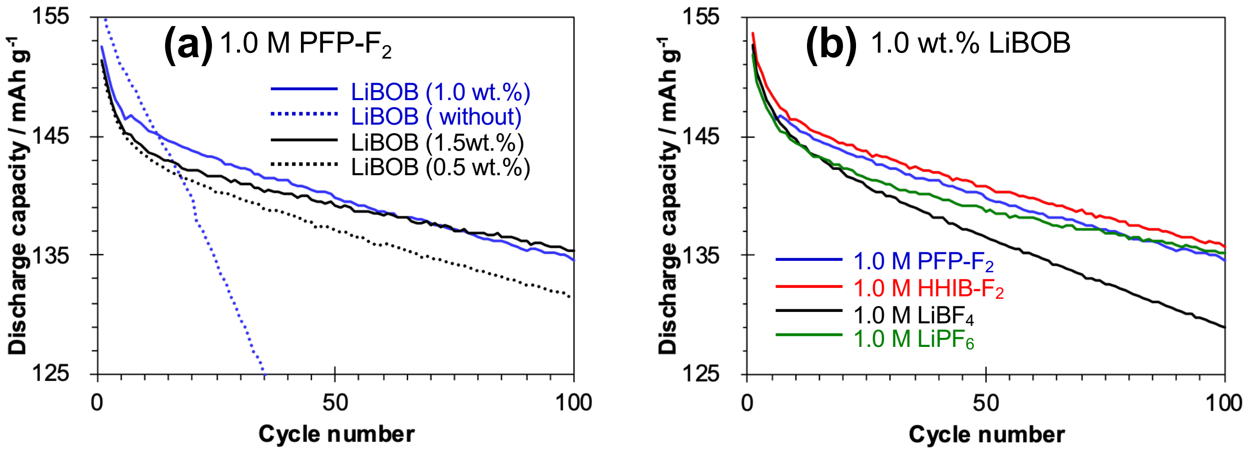

Figure S4. (a) Cycle performance of full cells with 1 M PFP-F_2 electrolyte and an additive LiBOB. The blue solid line is 1.0 wt.% LiBOB (Fig. 2b) and the blue dotted line is without LiBOB (Fig. 2b). The black solid line shows 1.5 wt.% of LiBOB and the black cotted line shows 0.5 wt.% of LiBOB. (60°C, 3 C rate within a voltage range of 3.0 V to 4.3 V) (b) Cycle performance compared to $LiPF_6$. The solid blue line shows the use of PFP-F_2 as an electrolyte, the solid red line also shows the use of HHIB-F_2, and the solid black line shows the use of $LiBF_4$ (Fig. 2b). And $LiPF_6$ electrolyte is the green line. All electrolyte solution contain LiBOB (1 wt.%).

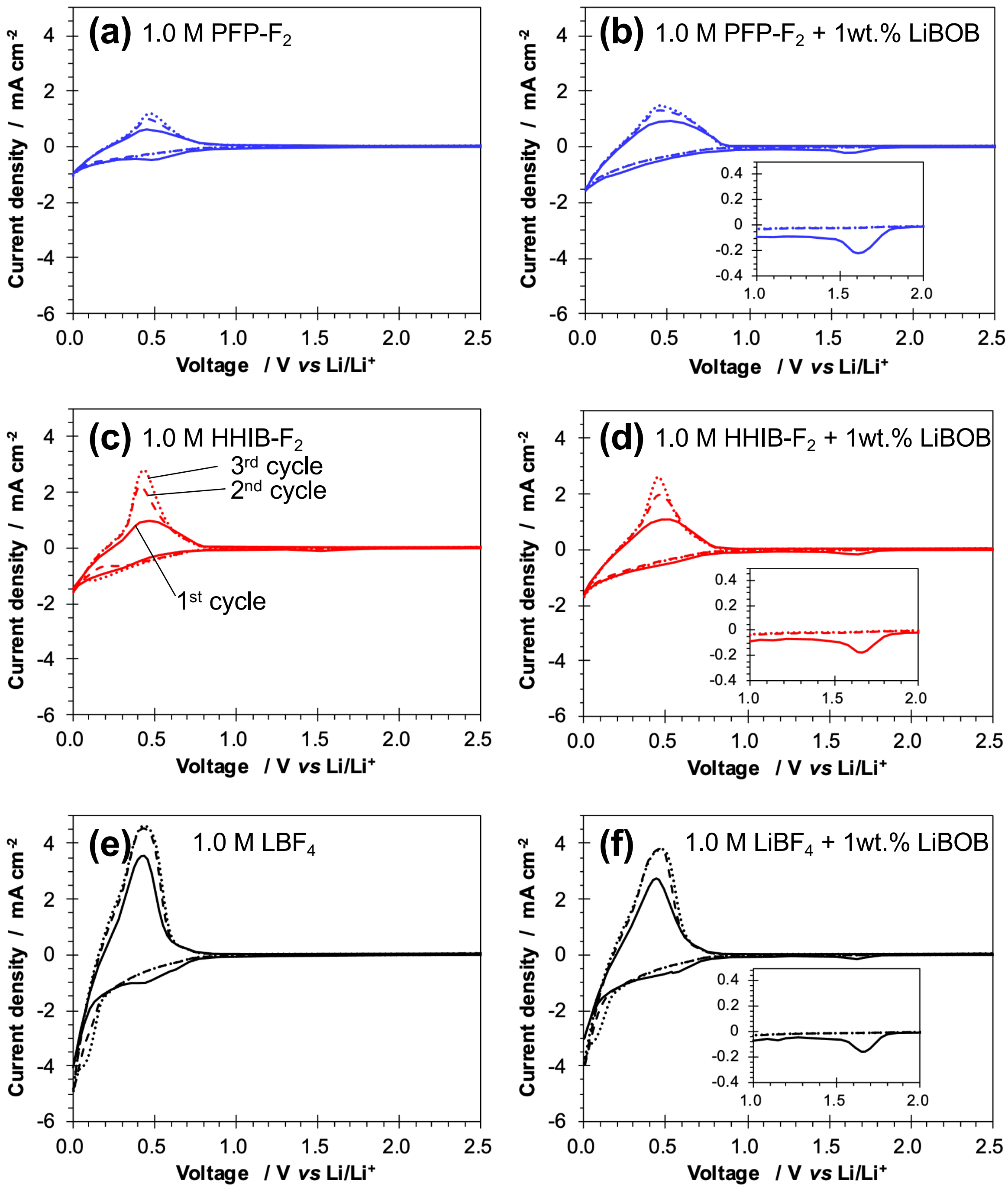

Figure S5. Cyclic voltammogram data (reduction side) of each electrolyte solutions at a scan rate of 1 mV s^{-1} at 25°C. Lithum half-cell was used and negative electrode (graphite) was used as working electrode. ()

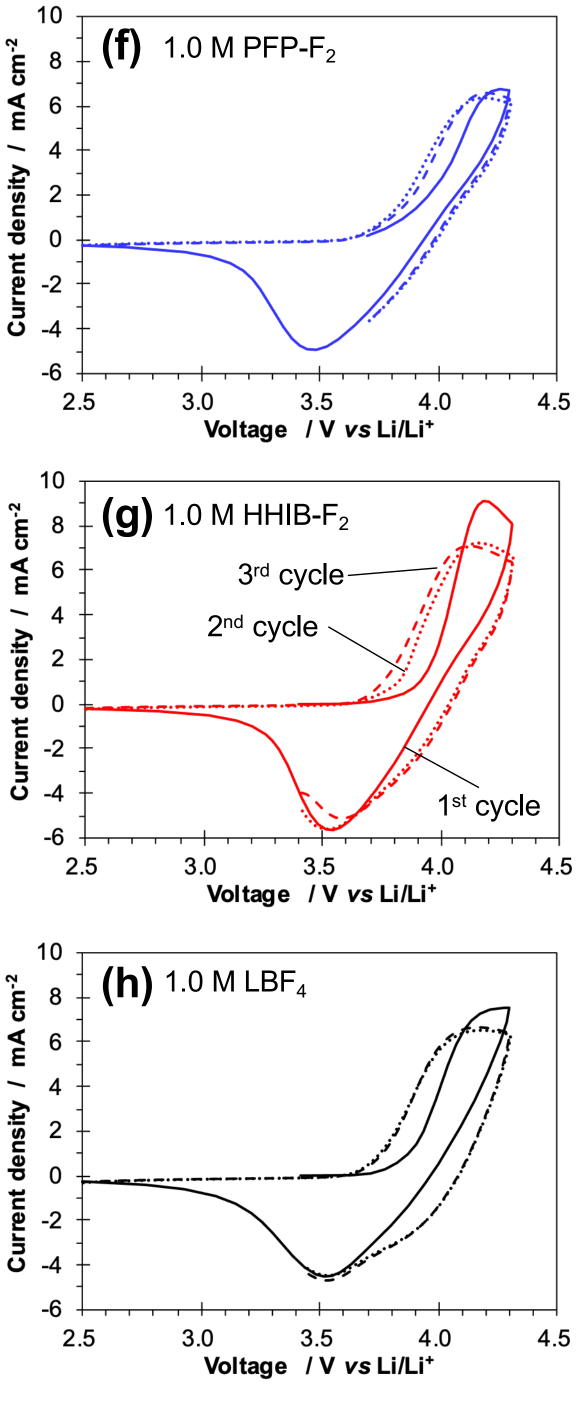

Figure S5. Cyclic voltammogram data (Oxidation side) of each electrolyte solutions at a scan rate of 1 mV s^{-1} at 25°C. Lithum half-cell was used and negative electrode (graphite) was used as working electrode.

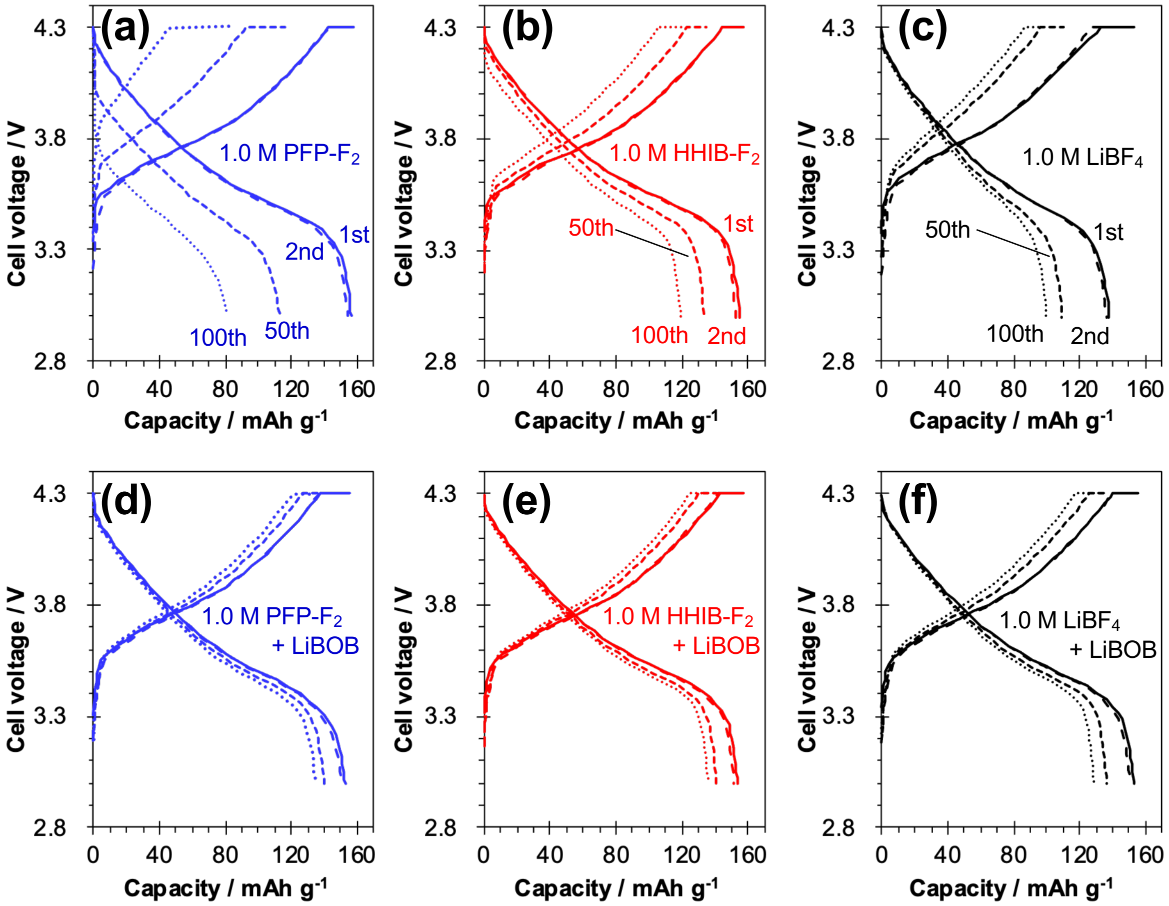

Figure S6. (a), (b), (c),(d),(e),(f) Charge/discharge curves of full cells with three different electrolyte salts. The blue shows PFP-F_2 and red shows HHIB-F_2, and the black shows $LiBF_4$. The upper column show the electrolyte solution without LiBOB, and the lower column show the electrolyte solution with 1 wt.% LiBOB. (60°C, 3 C rate within a voltage range of 3.0 to 4.3 V).

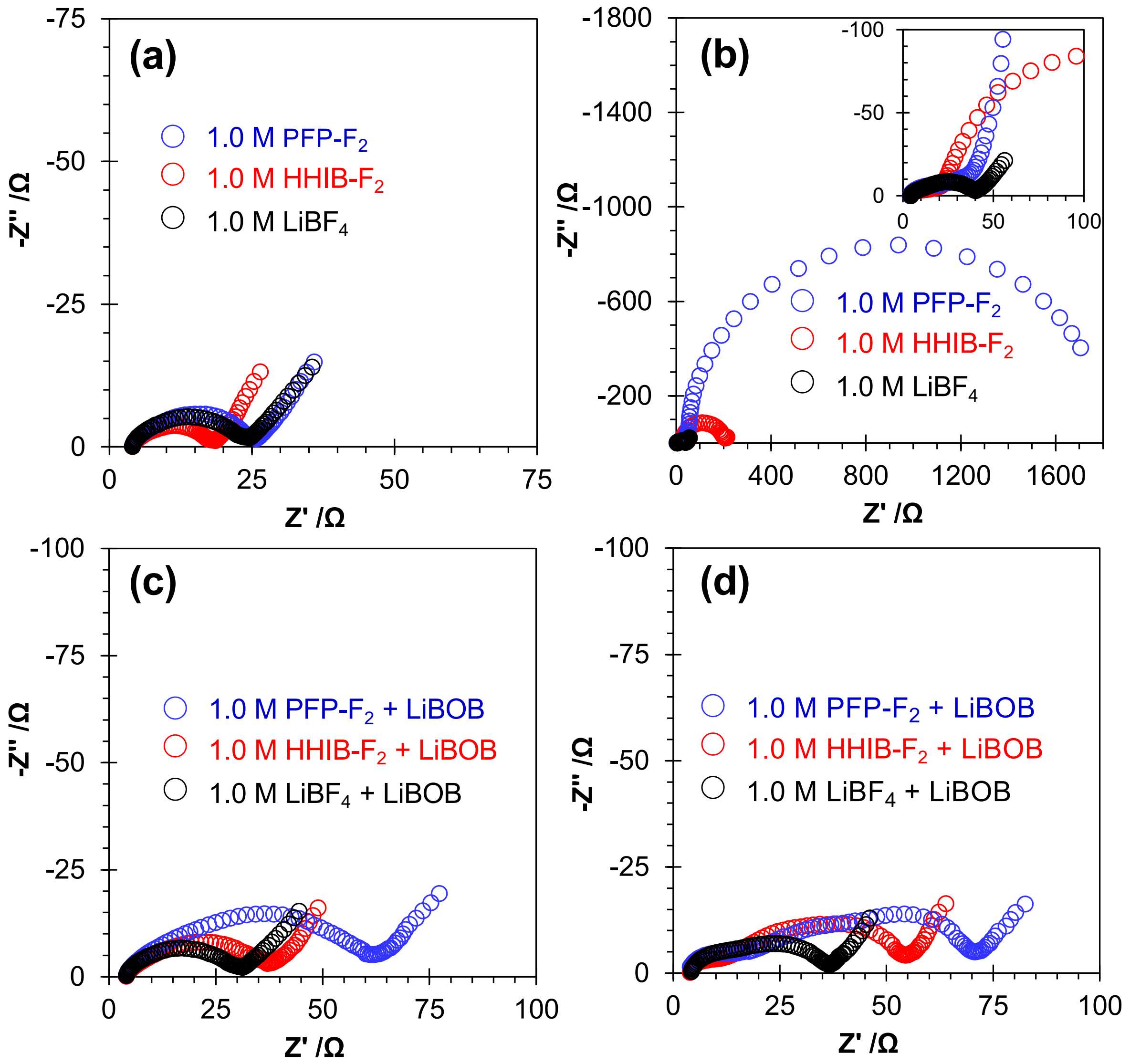

Figure S7. EIS date of full cells with three different electrolytes, **(a)** before cycle test (**Scheme S1**. EIS-1) without LiBOB, (**b**) after cycle test (**Scheme S1**.EIS-2) without LiBOB, (**c**) before cycle test with 1wt.% LiBOB, (**d**) after cycle test with 1wt.% LiBOB. Measurements were performed on cells with 100% SOC (after 0.2C charge at 25°C) at an amplitude of 10 mV and frequency of 100 kHz to 10 mHz at 25°C.

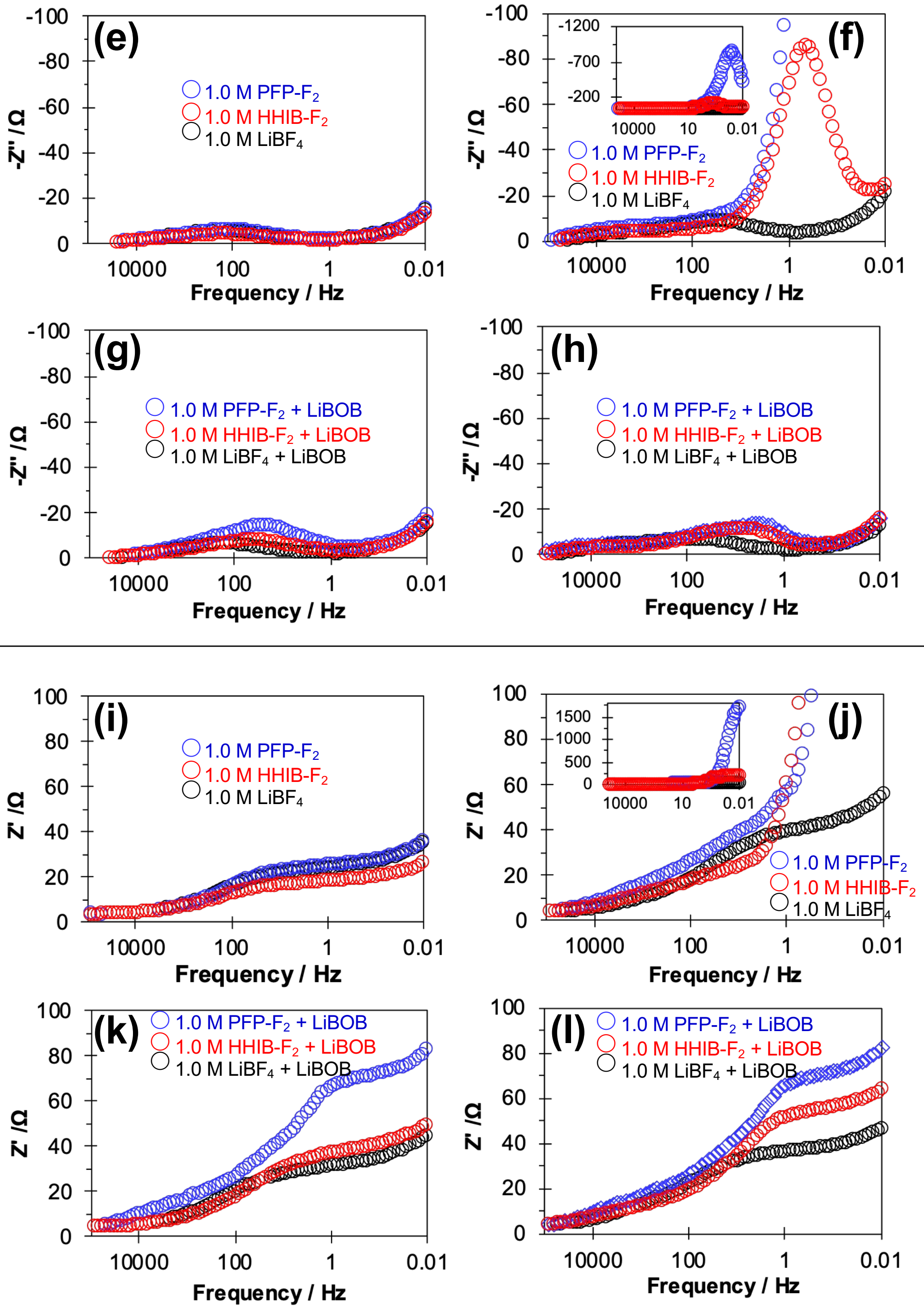

Figure S7. Bode plots graphs of EIS date of full cells with three different electrolytes, **(e), (i)** before cycle test (**Scheme S1**. EIS-1) without LiBOB, (**f**), (**j**) after cycle test (**Scheme S1**.EIS-2) without LiBOB, (**g**), (**k**) before cycle test with 1wt.% LiBOB, (**h**), (**l**) after cycle test with 1wt.% LiBOB. Measurements were performed on cells with 100% SOC (after 0.2C charge at 25°C) at an amplitude of 10 mV and frequency of 100 kHz to 10 mHz at 25°C.

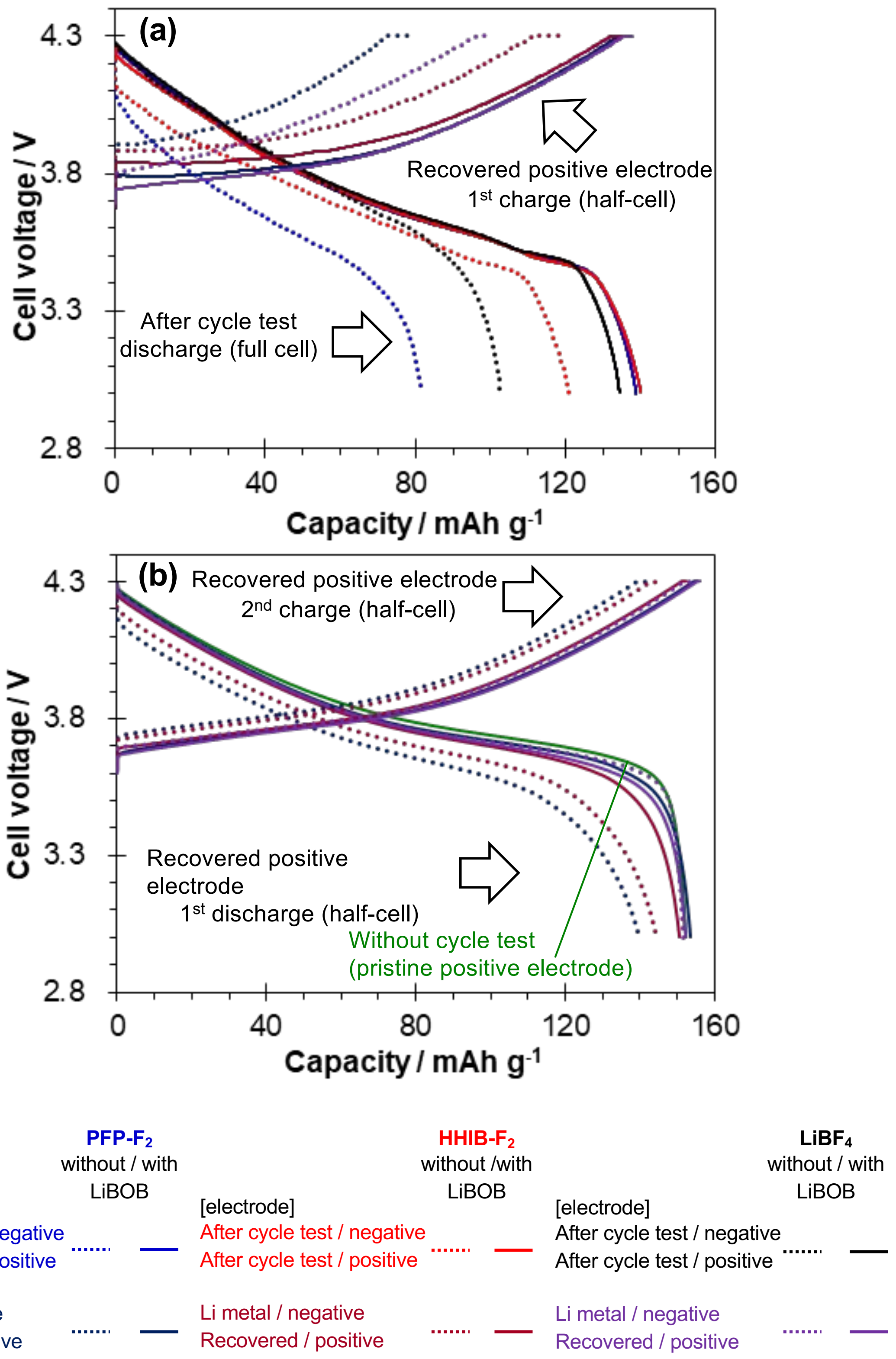

Figure S8. Charge and discharge curves for various systems. Blue and dark blue indicate PFP-F_2 as the electrolyte at cycle test. Red and dark red indicate HHIB-F_2, and black and violet indicate $LiBF_4$. A green line indicates a half-cell with pristine positive electrode and Li metal. The dotted lines are cells without LiBOB added in the cycle test. The solid lines show data of a cell using LiBOB. (a) Comparison of discharge capacity after cycle test and 1st charge capacity of Li half-cell using recovered positive electrode. (25°C, 0.2 C within a voltage range of 3.0 V to 4.3 V) (b) Comparison of 1st discharge and 2nd charge capacity with recovered positive electrode and Li metal negative electrode together with a discharge capacity of half-cell with pristine positive electrode and Li metal negative electrode. (25°C, 0.2 C within a voltage range of 3.0 V to 4.3 V)

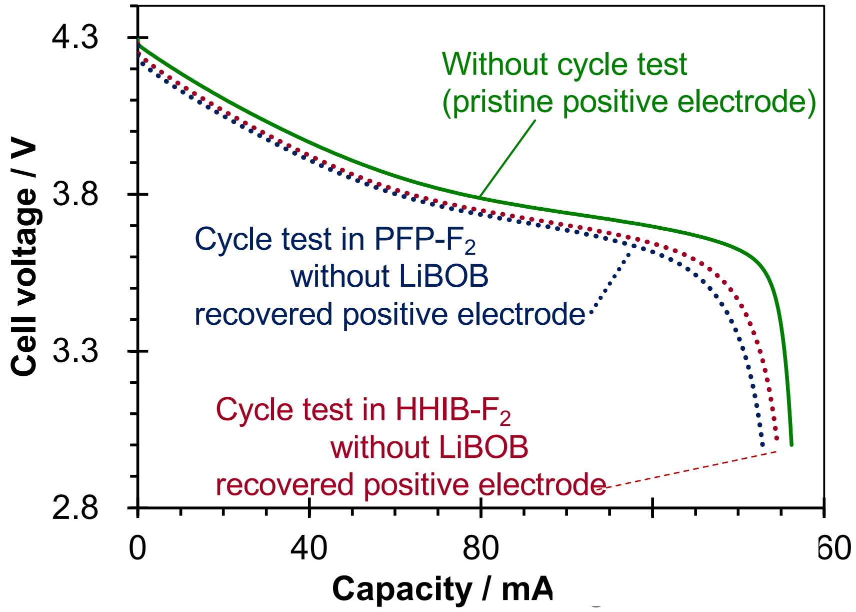

Figure S9. Discharge capacity of the recovered positive electrode was checked using Li metal and 1.0 M $LiBF_4$ EC/EMC (1/2 v) solution. The solid green line shows the discharge curve of the pristine positive electrode, dark blue dotted line indicates PFP-F_2, and dark red dotted line indicates HHIB-F_2. (25°C, 0.1 C within a voltage range of 3.0 to 4.3 V)

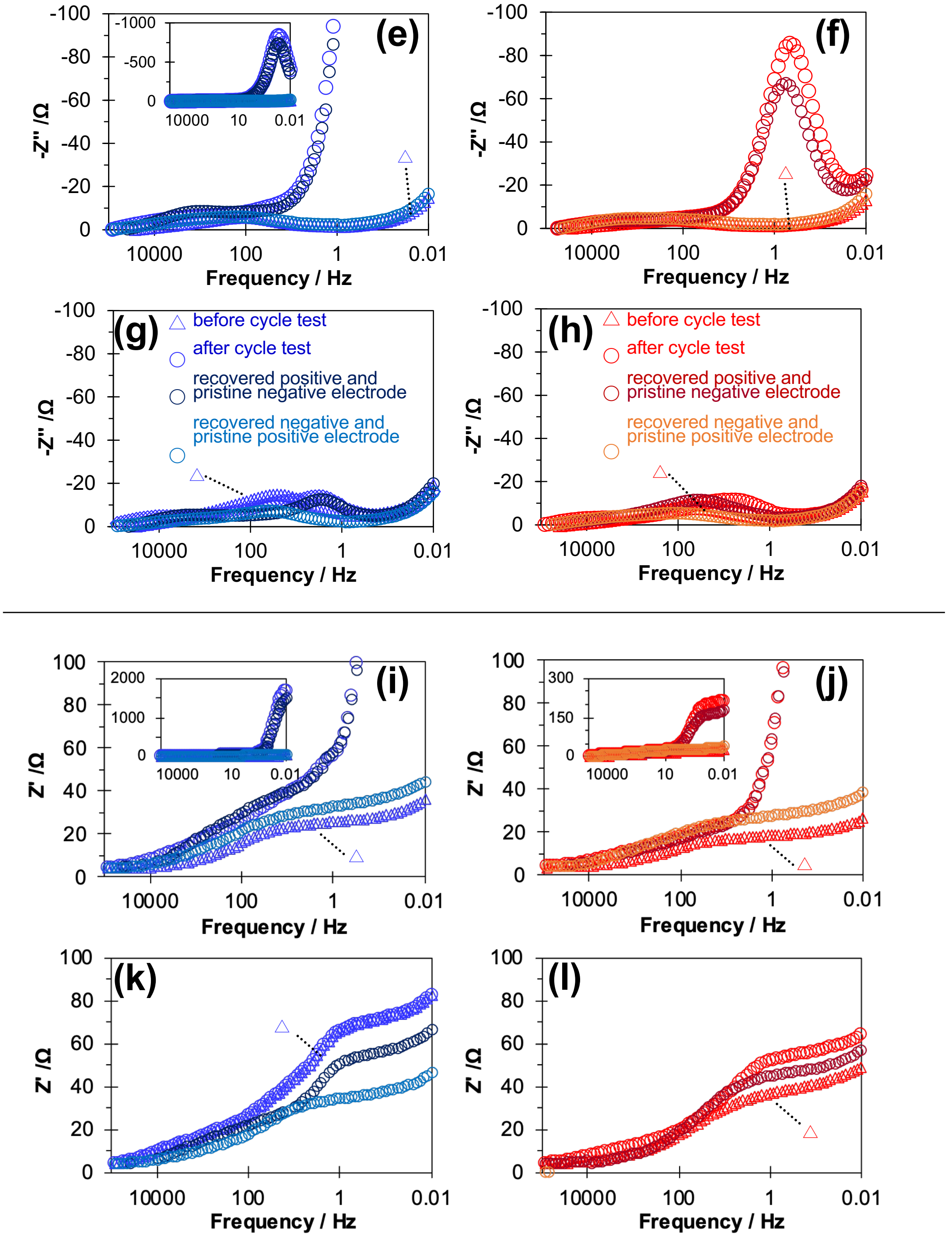

Figure S10. Bode plots graphs of EIS date in Fig. 5 with recovered and pristine electrodes in (**e**), (**i**) PFP-F_2, (**f**), (**j**) HHIB-F_2, (**g**), (**k**) PFP-F_2 with LiBOB, and (**h**), (**l**) HHIB-F_2 with LiBOB. Each legend represents the conditions of the cell for EIS measurements. Measurements were performed on cells with 100% SOC (after 0.2 C charge at 25°C) at an amplitude of 10 mV and frequency of 100 kHz to 10 mHz at 25°C.

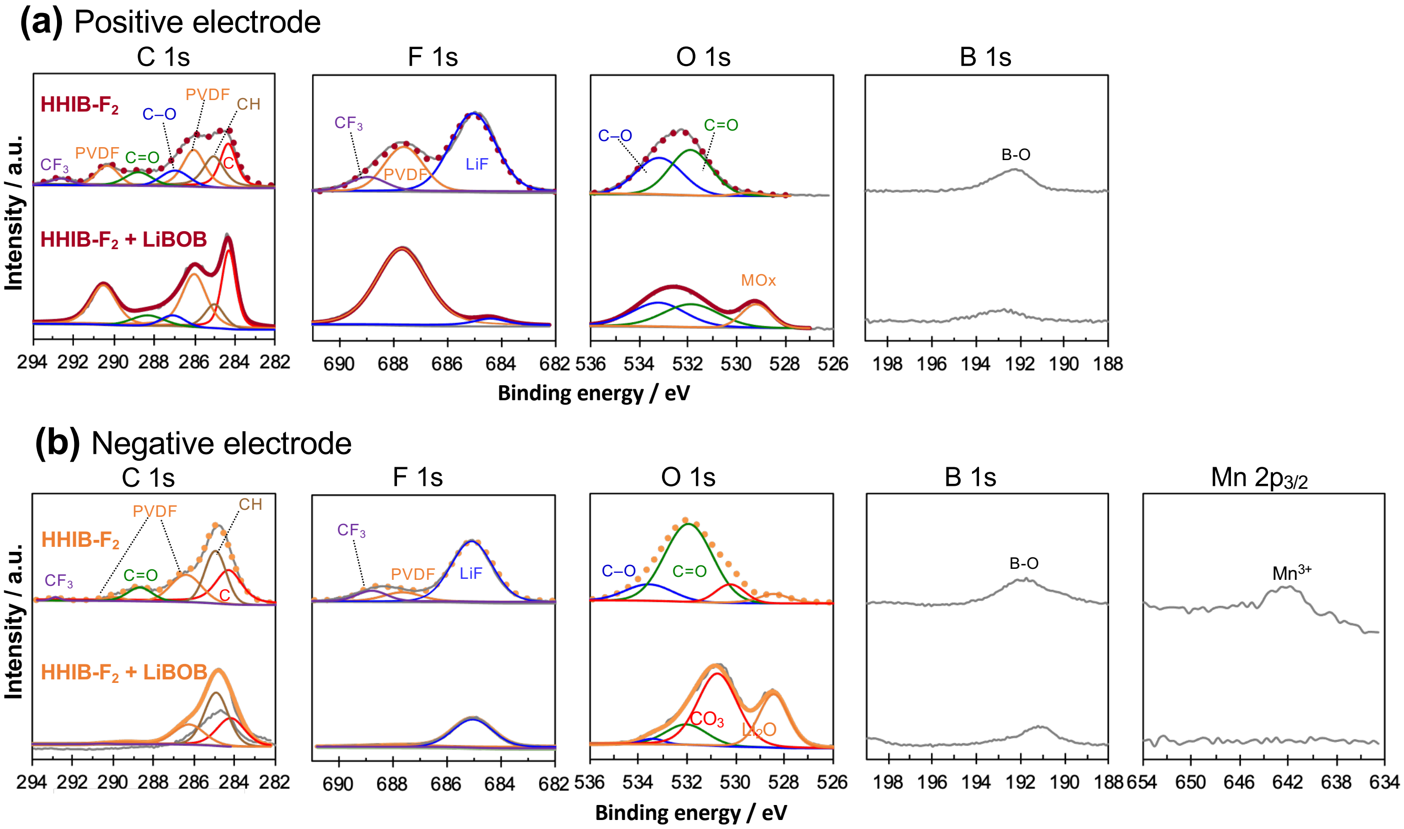

Figure S11. XPS spectra of recovered (a) positive and (b) negative electrode surface after the cycle test in HHIB-F_2 electrolyte with and without LiBOB. (Recovered electrodes were recovered from the cells, washed, and dried under reduced pressure before XPS measurement.) From left to right, the photoemission lines for the C 1s, F 1s, O 1s,B 1s, and Mn $2p_{3/2}$. All spectra were calibrated with the adventitious hydrocarbons at 284.3 eV and background corrected using a Shirley background. The cycle tests for recovered electrodes were performed at 60°C, 3 C within a voltage range of 3.0 to 4.3 V in 1.0 M HHIB-F_2 in EC/EMC with and without 1wt.% LiBOB.

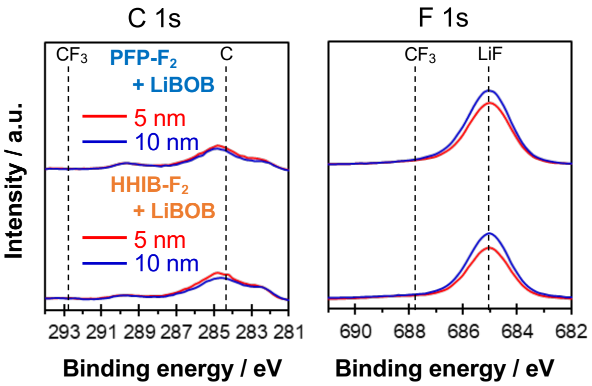

Figure S12. XPS spectra of the recovered negative electrode surface after the cycle test, where the surface was etched using argon gas cluster ion beam; red lines show the etching depth of 5 nm and blue lines show 10 nm.

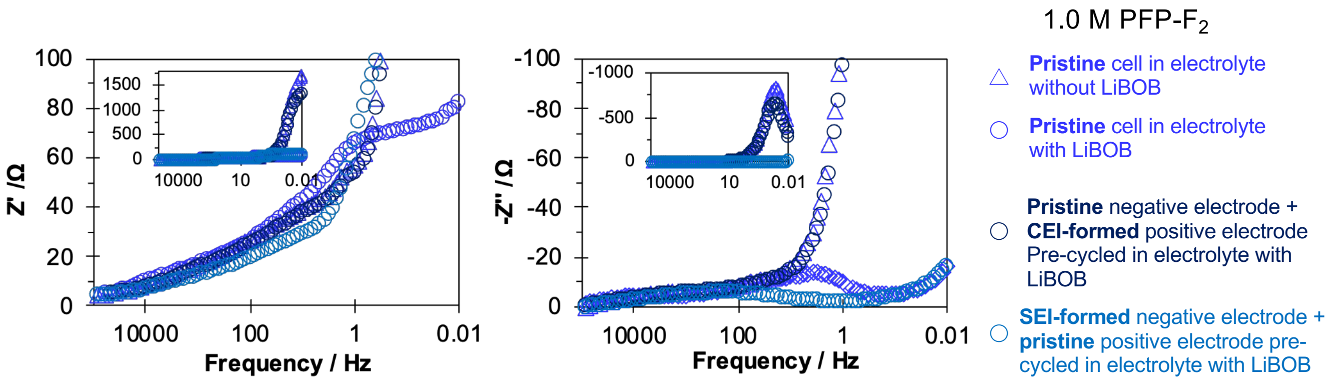

Figure S13. Bode plot of EIS data (Fig. 8b) after cycle test (corresponds to the EIS-2 in **Scheme S1** and EIS-5 in **Scheme S4**) of four different full cells. For comparison, the results of pristine cells with LiBOB (same data as the blue circles in Figure S7h, 7l) and without LiBOB (same data as the blue circles in Figure S7f, 7j) are shown as blue circles and triangles. Measurements were performed on cells with 100% SOC (after 0.2C charge at 25°C) at an amplitude of 10 mV and frequency of 100 kHz to 10 mHz at 25°C.

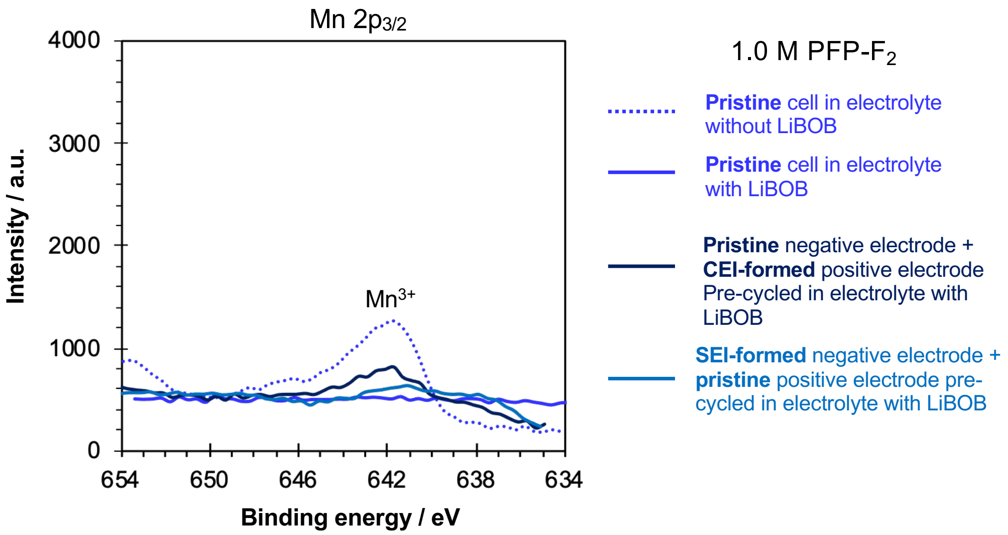

Figure S14. XPS spectra (Mn $2p_{3/2}$) of four types of recovered negative electrode surface after the cycle test in HHIB-F_2 electrolyte. The pristine cell data (blue solid and dotted line) is the same as in FIg.6b. The light blue solid line is the data of the negative electrode after the cycle test using the negative electrode with SEI formed by LiBOB. And the dark blue solid line is the data of the negative electrode after the cycle test using the positive electrode with CEI formed by LiBOB.
